# Supplementary material for: 3,4-Seco-Isopimarane Diterpenes from the Twigs and Leaves of Isodon Flavidus
Source: Molecules. 2022 May 12;27(10):3098. doi: 10.3390/molecules27103098 (PMC9143206; doi:10.3390/molecules27103098)
Supplement: Supplementary file 1 [file molecules-27-03098-s001.zip › molecules-1677754-supplementary.pdf]

Supporting Information

Isopimarane diterpenoids from the twigs and leaves of *Isodon flavidus*

Wan-Fei Li [1,2,†], Zheng-Ming Liang [2,†], Chen-Liang Zhao [1,2], Nga Yi Tsang [2], Ji-Xin Li [1], Ya-Hua Liu [1] , Kang He [1], Lu-Tai Pan [1], Lijun Rong [3], Juan Zou [1]* and Hong-Jie Zhang [2]*

[1] College of Pharmacy, Guizhou University of Traditional Chinese Medicine, Dongqing South Road, Guiyang city, Guizhou Province 550025, People's Republic of China; li_wanfei@163.com (W.-F.L.); 18482082@life.hkbu.edu.hk (C.-L. Z.); lijixinmylove@yeah.net (J.-X. L.); lyh_1123@126.com (Y.-H. L.); hekang0851@163.com (K. H.); ltpan@sina.cn (L.-T. P.);

[2] School of Chinese Medicine, Hong Kong Baptist University, Hong Kong SAR, P. R. China; 18482473@life.hkbu.edu.hk (Z.-M.L.); 12012173@life.hkbu.edu.hk (N.Y.T.)

[3] Department of Microbiology and Immunology, College of Medicine, University of Illinois at Chicago, 909 South Wolcott Avenue, Chicago, IL 60612, United States; lijun@uic.edu (L.R.)

* Correspondence: zoujuan466@gzy.edu.cn (J. Z.); zhanghj@hkbu.edu.hk (H.-J.Z.); Tel.: +852-34112956 (H.-J.Z.)

† These authors contributed equally to this work.

Table of Contents

X-ray analysis of fladin B (1) 4

Figure S1. The UV spectrum of fladin B (**1**) in $CHCl_3$. 5

Figure S2. The IR (KBr disc) spectrum of fladin B (**1**). 6

Figure S3. The HR-ESI-MS spectrum of fladin B (**1**). 7

Figure S4. ^{1}H NMR spectrum of fladin B (**1**) in $CDCl_3$ (400 MHz). 8

Figure S5. ^{13}C NMR spectrum of fladin B (**1**) in $CDCl_3$ (100 MHz). 9

Figure S6. DEPT spectra of fladin B (**1**) in $CDCl_3$. 10

Figure S7. HSQC spectrum of fladin B (**1**) in $CDCl_3$. 11

Figure S8. HMBC spectrum of fladin B (**1**) in $CDCl_3$. 12

Figure S9. ^{1}H-^{1}H COSY spectrum of fladin B (**1**) in $CDCl_3$. 13

Figure S10. NOESY spectrum of fladin B (**1**) in $CDCl_3$. 14

Figure S11. The UV spectrum of fladin C (**2**) in $CHCl_3$. 15

Figure S12. The IR (KBr disc) spectrum of fladin C (**2**). 16

Figure S13. The HR-ESI-MS spectrum of fladin C (**2**). 17

Figure S14. ^{1}H NMR spectrum of fladin C (**2**) in $CDCl_3$ (500 MHz). 18

Figure S15. ^{13}C NMR spectrum of fladin C (**2**) in $CDCl_3$ (125 MHz). 19

Figure S16. DEPT spectra of fladin C (**2**) in $CDCl_3$. 20

Figure S17. HSQC spectrum of fladin C (**2**) in $CDCl_3$. 21

Figure S18. HMBC spectrum of fladin C (**2**) in $CDCl_3$. 22
Figure S19. ^1H-^1H COSY spectrum of fladin C (**2**) in $CDCl_3$. 23
Figure S20. NOESY spectrum of fladin C (**2**) in $CDCl_3$. 24
Figure S21. The UV spectrum of fladin D (**3**) in $CHCl_3$. 25
Figure S22. The IR (KBr disc) spectrum of fladin D (**3**). 26
Figure S23. The HR-ESI-MS spectrum of fladin D (**3**). 27
Figure S24. ^1H NMR spectrum of fladin D (**3**) in $CDCl_3$ (400 MHz). 28
Figure S25. ^{13}C NMR spectrum of fladin D (**3**) in $CDCl_3$ (100 MHz). 29
Figure S26. DEPT spectra of fladin D (**3**) in $CDCl_3$. 30
Figure S27. HSQC spectrum of fladin D (**3**) in $CDCl_3$. 31
Figure S28. HMBC spectrum of fladin D (**3**) in $CDCl_3$. 32
Figure S29. ^1H-^1H COSY spectrum of fladin D (**3**) in $CDCl_3$. 33
Figure S30. NOESY spectrum of fladin D (**3**) in $CDCl_3$. 34

X-ray analysis of fladin B (1)

Fladin B (**1**) was obtained as single crystals by slow evaporation from MeOH solution of the pure compound at room temperature. Crystallographic data were collected on a Bruker *APEX*-II CCD instrument using Cu Kα radiation. Cell refinement and data reduction were accomplished with the aid of a Bruker SAINT, whereas structure was solved by means of SHELXTL [34,35]. The crystallographic data for **1** (CCDC 1033449) can be obtained on request from the Cambridge Crystallographic Data Centre via www.ccdc.cam.ac.uk/data_request/cif.

Fladin B: $C_{20}H_{30}O_3$, M = 318.44, monoclinic, a = 11.4575(12) Å, b = 6.3205(6) Å, c = 12.5407(13) Å, α = 90.00°, β = 108.004(4)°, γ = 90.00°, V = 863.69(15) Å^3, T = 100(2) K, space group P21, Z = 2, μ(CuKα) = 0.632 mm^{-1}, 4892 reflections measured, 2556 independent reflections (R_{int} = 0.0574). The final R_1 values were 0.0936 ($I > 2\sigma(I)$). The final $wR(F^2)$ values were 0.2355 ($I > 2\sigma(I)$). The final R_1 values were 0.0948 (all data). The final $wR(F^2)$ values were 0.2382 (all data). The goodness of fit on F^2 was 1.108. Flack parameter = -0.1(4). The Hooft parameter is 0.10(15) for 917 Bijvoet pairs.

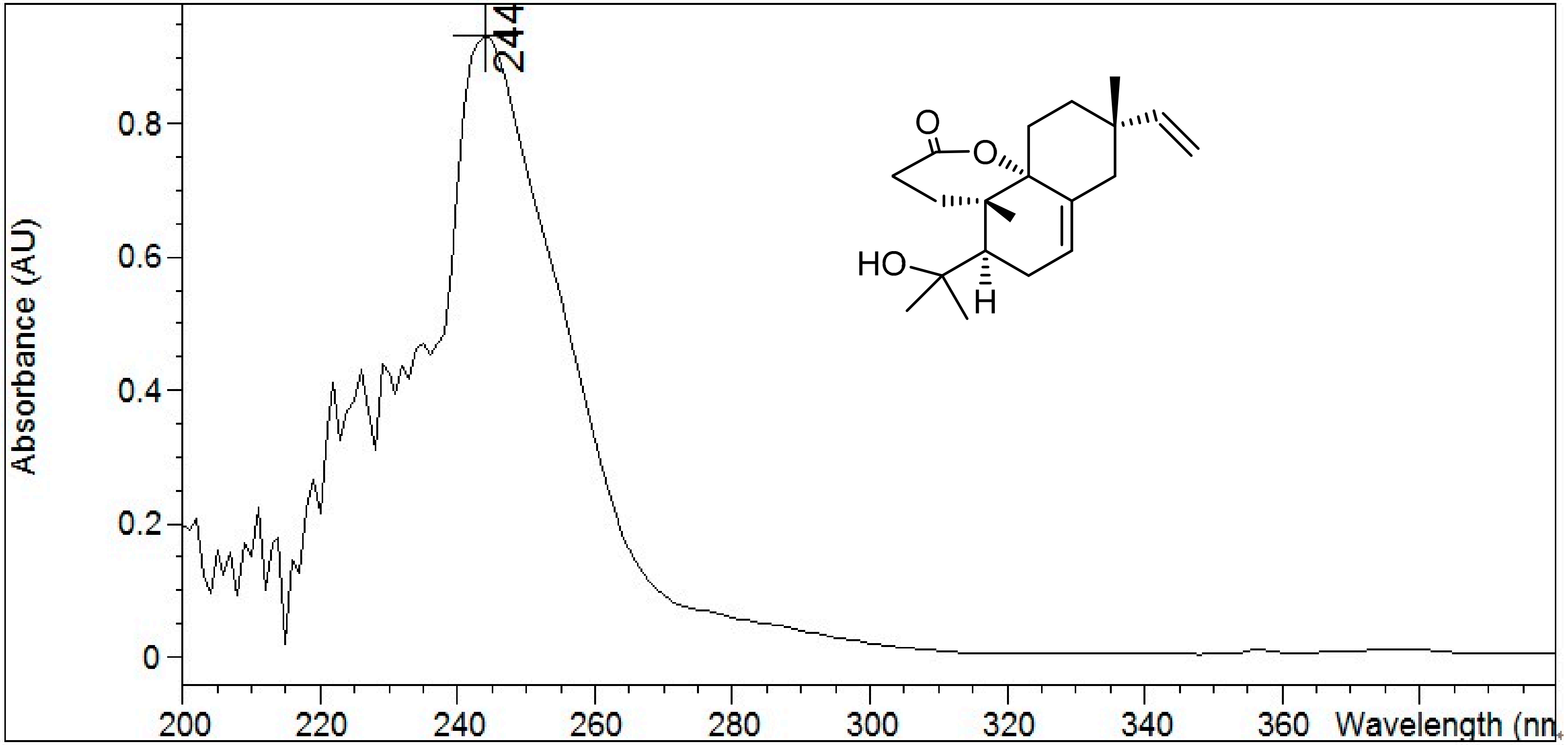

#	Name	Peaks(nm)	Abs(AU)
1	IZ-13	244.0	0.93358

1.

Figure S1. The UV spectrum of fladin B (**1**) in $CHCl_3$.

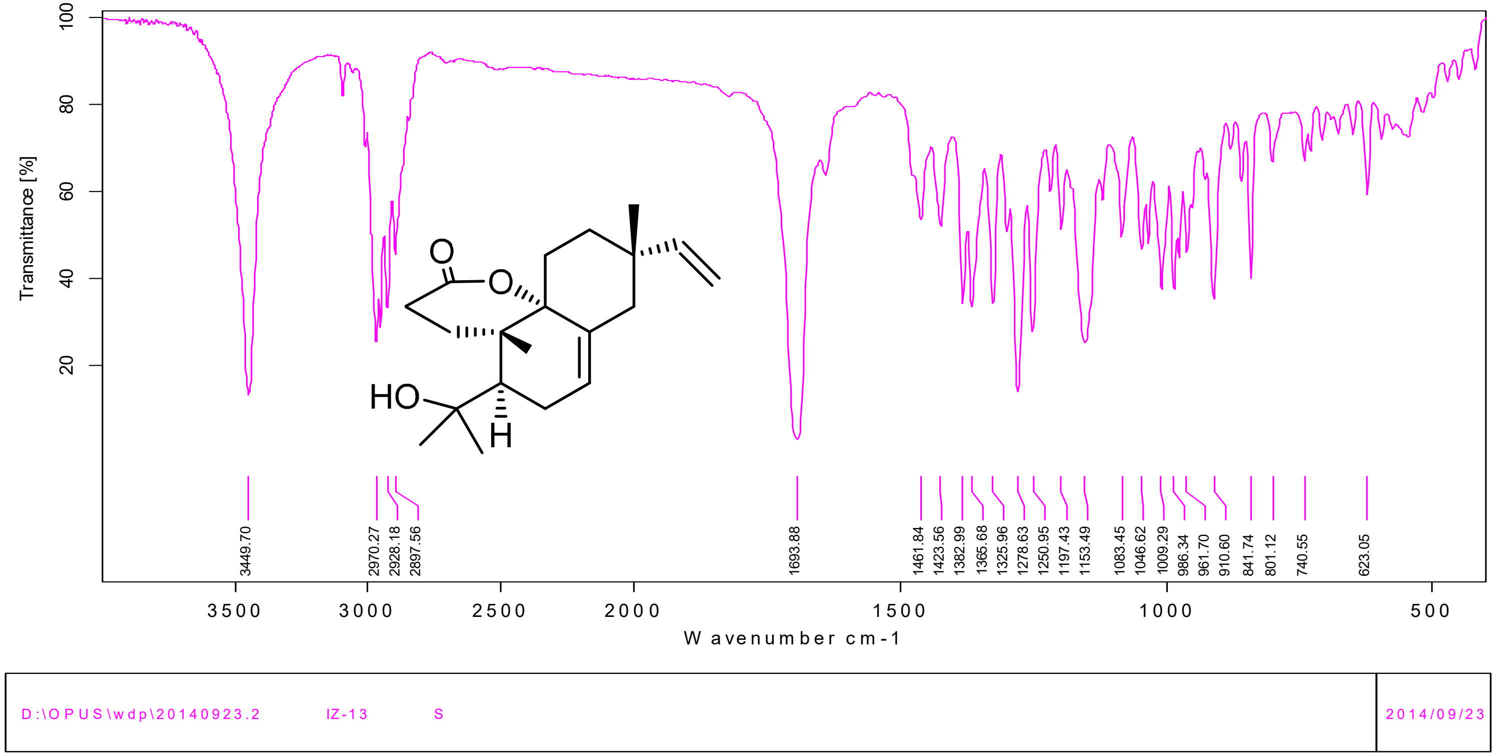

Figure S2. The IR (KBr disc) spectrum of fladin B (**1**).

Mass Spectrum SmartFormula Report

Analysis Info

Analysis Name	D:\Data\Zhang HJ\LKL\IZ-13_1-D,1_01_12813.d	Acquisition Date	10/17/2014 3:21:04 PM
Method	wide_pos (13-05-20).m	Operator	CMED
Sample Name	IZ-13	Instrument / Ser#	micrOTOF-Q 19
Comment			

Acquisition Parameter

Source Type	ESI	Ion Polarity	Positive	Set Nebulizer	2.5 Bar
Focus	Not active			Set Dry Heater	180 °C
Scan Begin	50 m/z	Set Capillary	4500 V	Set Dry Gas	8.0 l/min
Scan End	1600 m/z	Set End Plate Offset	-500 V	Set Divert Valve	Source

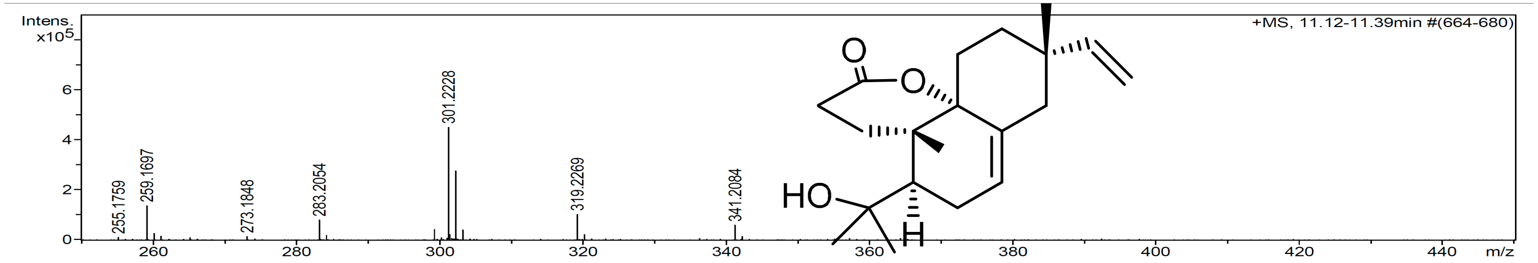

Meas. m/z	#	Formula	m/z	err [ppm]	Mean err [ppm]	rdb	N-Rule	e⁻ Conf	mSigma	Std I	Std Mean m/z	Std I VarNorm	Std m/z Diff	Std Comb Dev
319.2269														
	1	C 20 H 31 O 3	319.2268	-0.4	0.0	5.5	ok	even	6.34	0.0111	0.0003	0.0046	0.0006	0.8427
	2	C 16 H 27 N 6 O	319.2241	-8.8	-8.4	6.5	ok	even	9.50	0.0185	0.0027	0.0083	0.0005	0.9982
341.2084														
	1	C 20 H 30 Na O 3	341.2087	1.0	1.4	5.5	ok	even	7.20	0.0108	0.0006	0.0076	0.0008	0.6432
	2	C 18 H 25 N 6 O	341.2084	0.2	0.5	9.5	ok	even	10.60	0.0167	0.0003	0.0119	0.0007	0.6689
	3	C 17 H 29 N 2 O 5	341.2071	-3.7	-3.4	4.5	ok	even	13.07	0.0251	0.0012	0.0114	0.0008	0.7719
	4	C 16 H 26 N 6 Na O	341.2060	-6.9	-6.5	6.5	ok	even	15.27	0.0305	0.0022	0.0162	0.0007	0.8749
	5	C 22 H 29 O 3	341.2111	8.1	8.4	8.5	ok	even	16.24	0.0237	0.0029	0.0112	0.0008	0.8638
	6	C 15 H 30 N 2 Na O 5	341.2047	-10.8	-10.4	1.5	ok	even	25.29	0.0476	0.0036	0.0201	0.0008	0.9299
	7	C 13 H 25 N 8 O 3	341.2044	-11.6	-11.3	5.5	ok	even	26.01	0.0502	0.0039	0.0215	0.0006	0.9398

Bruker Compass DataAnalysis 4.0 printed: 10/21/2014 1:24:37 PM Page 1 of 1

Figure S3. The HR-ESI-MS spectrum of fladin B (**1**).

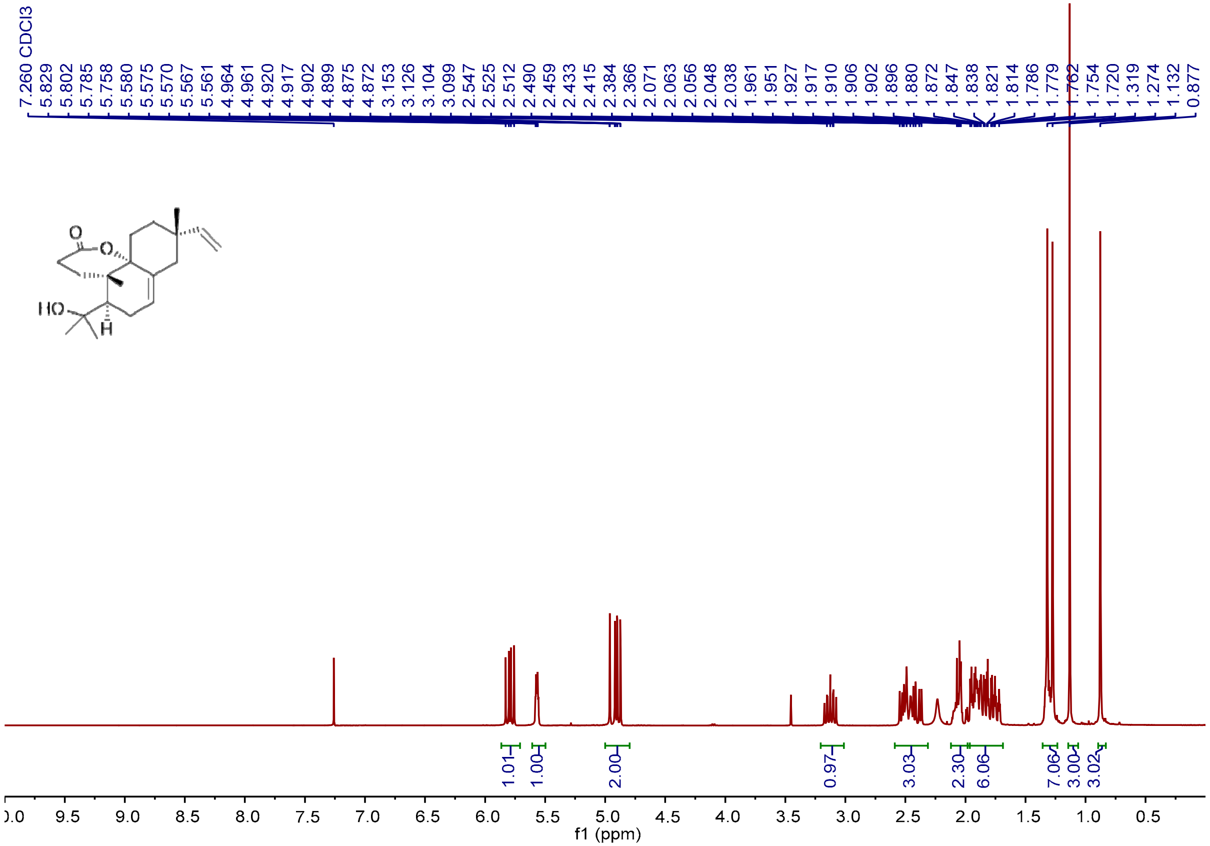

Figure S4. ^{1}H NMR spectrum of fladin B (**1**) in $CDCl_3$ (400 MHz).

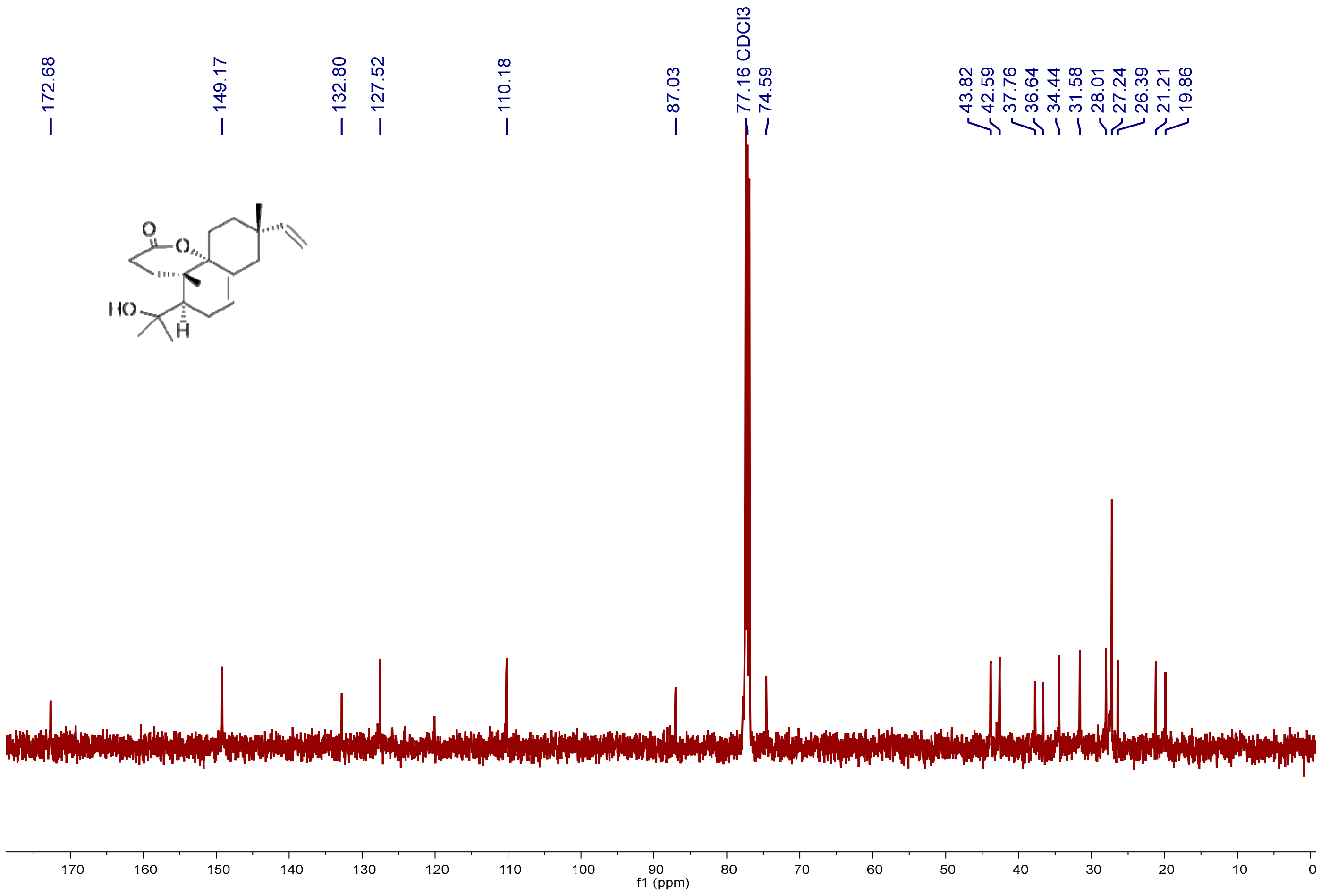

Figure S5. ^{13}C NMR spectrum of fladin B (**1**) in $CDCl_3$ (100 MHz).

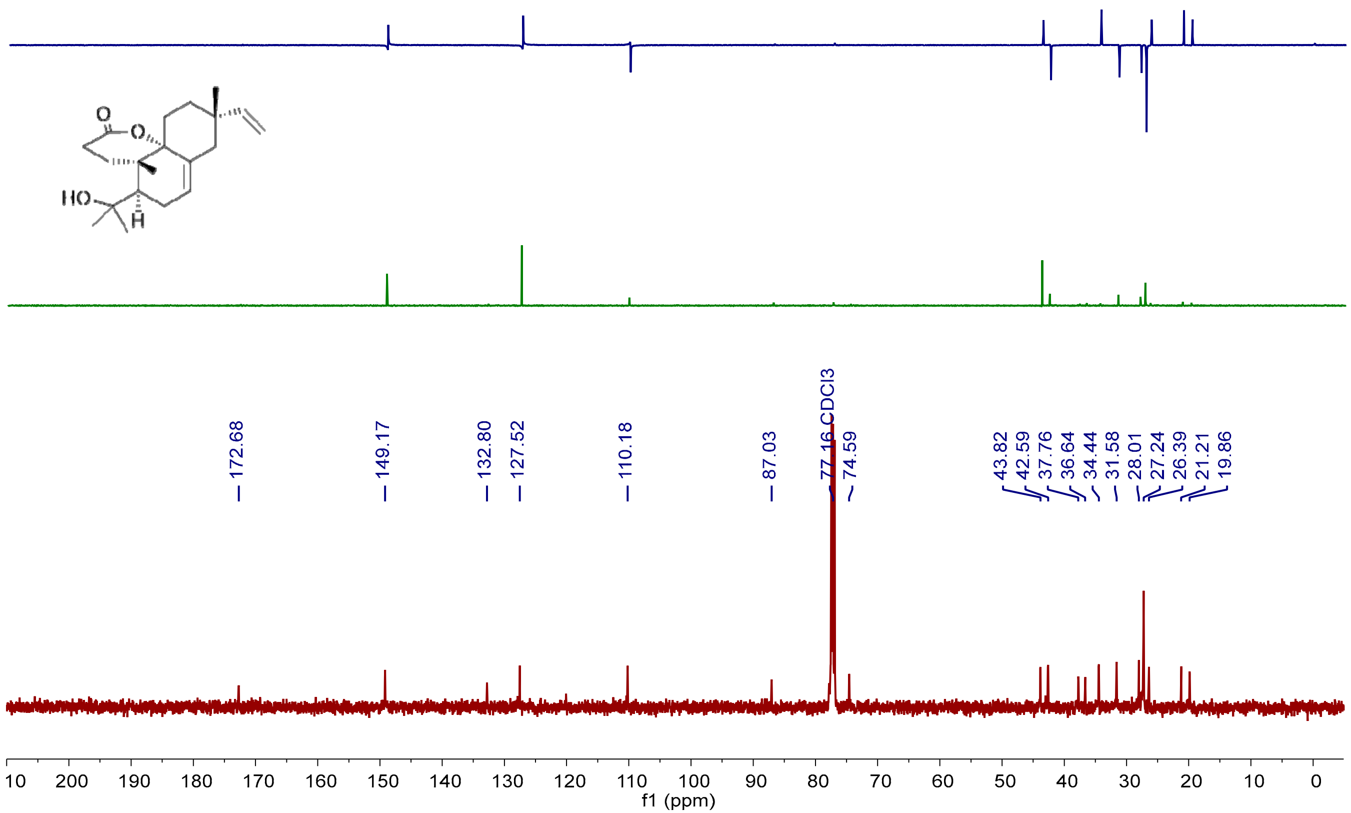

Figure S6. DEPT spectra of fladin B (**1**) in $CDCl_3$.

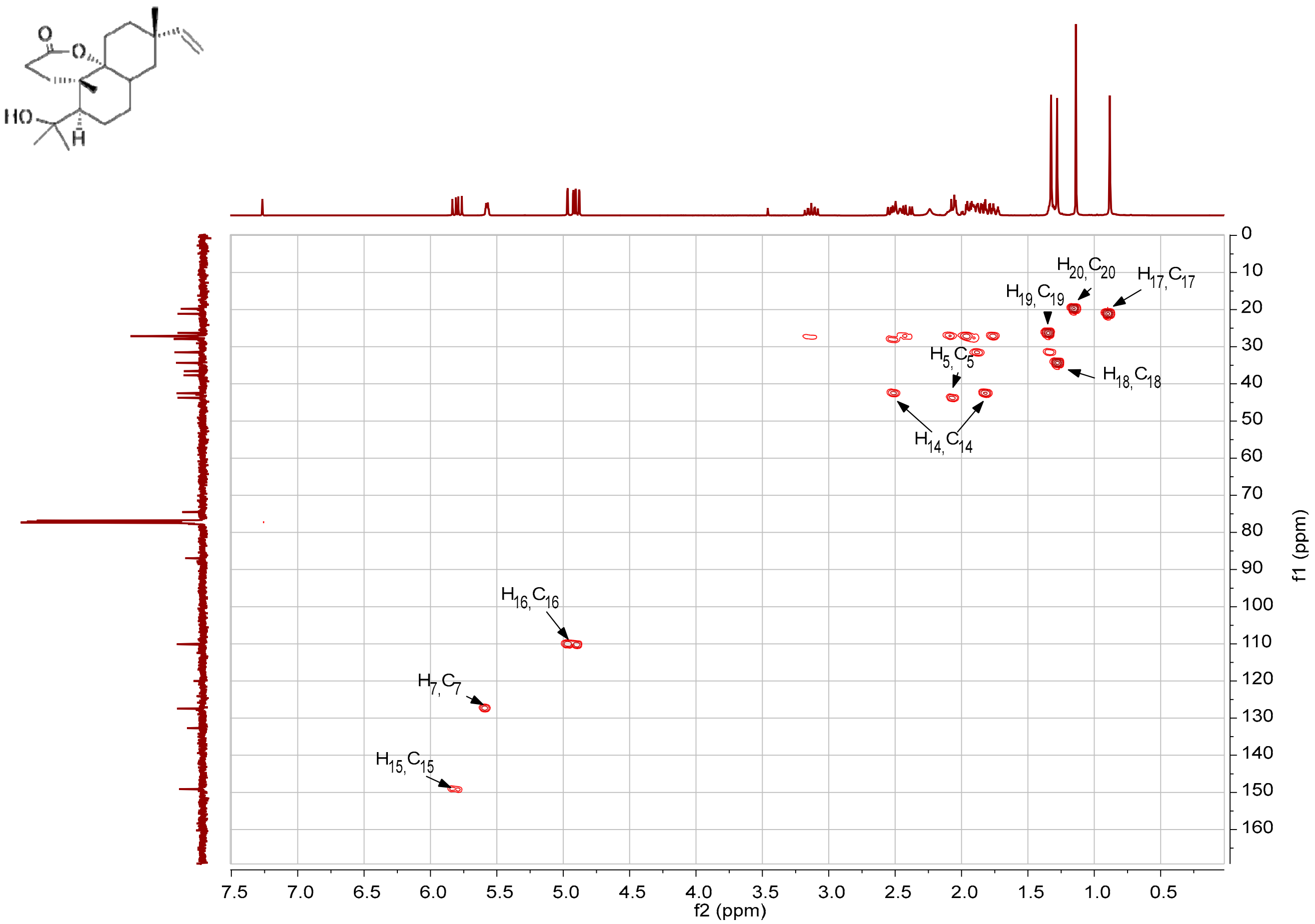

Figure S7. HSQC spectrum of fladin B (**1**) in $CDCl_3$.

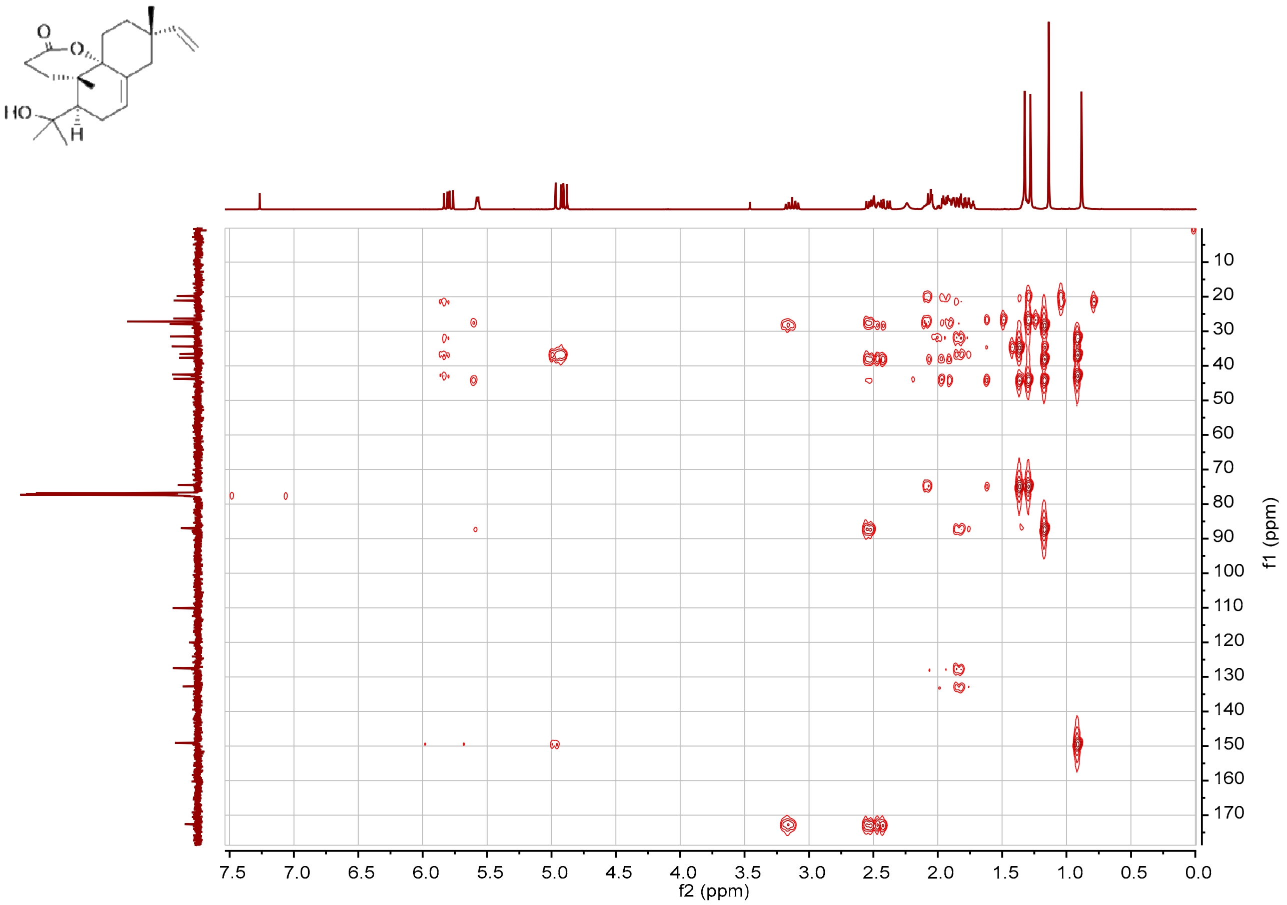

Figure S8. HMBC spectrum of fladin B (**1**) in $CDCl_3$.

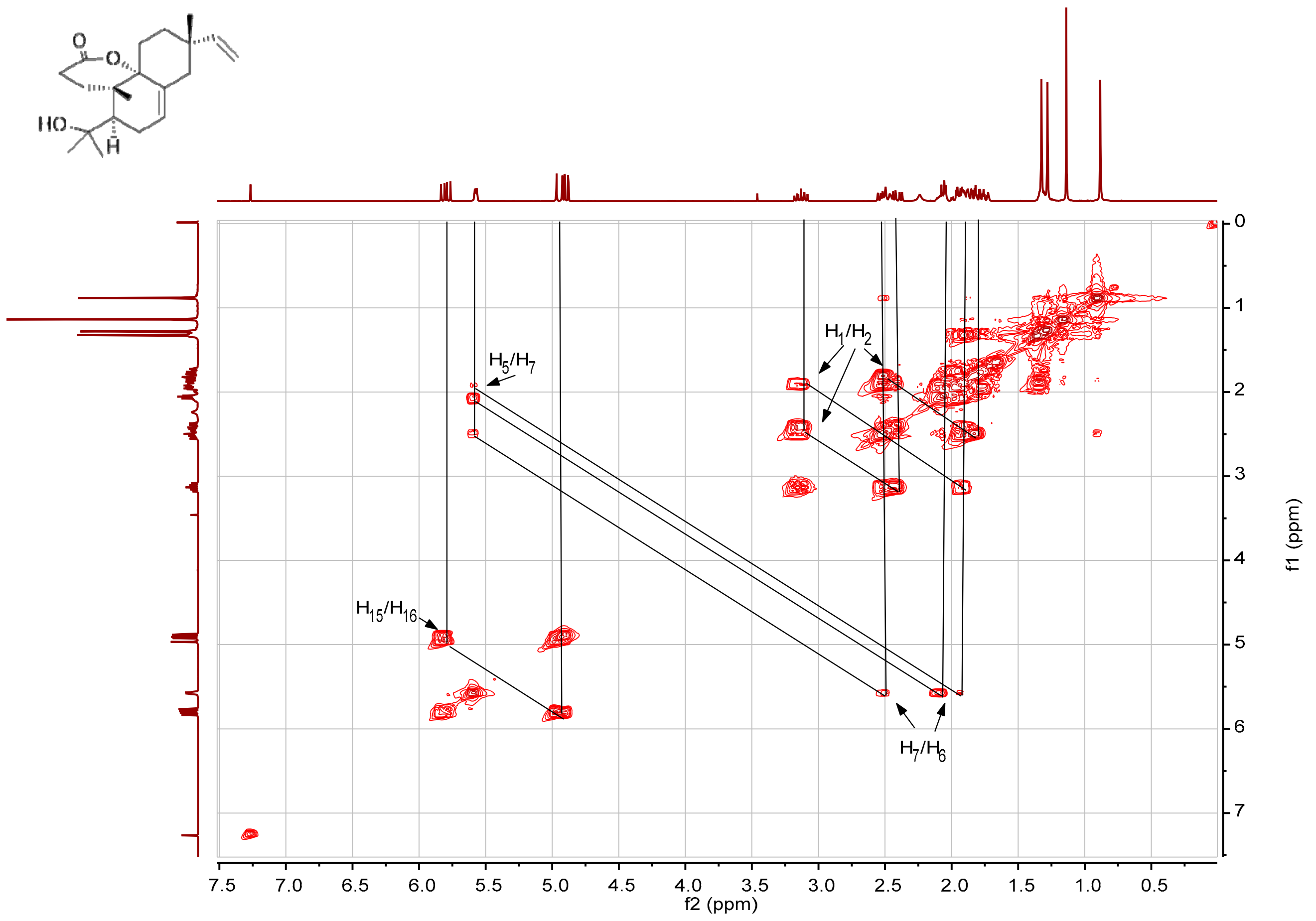

Figure S9. ^{1}H-^{1}H COSY spectrum of fladin B (**1**) in $CDCl_3$.

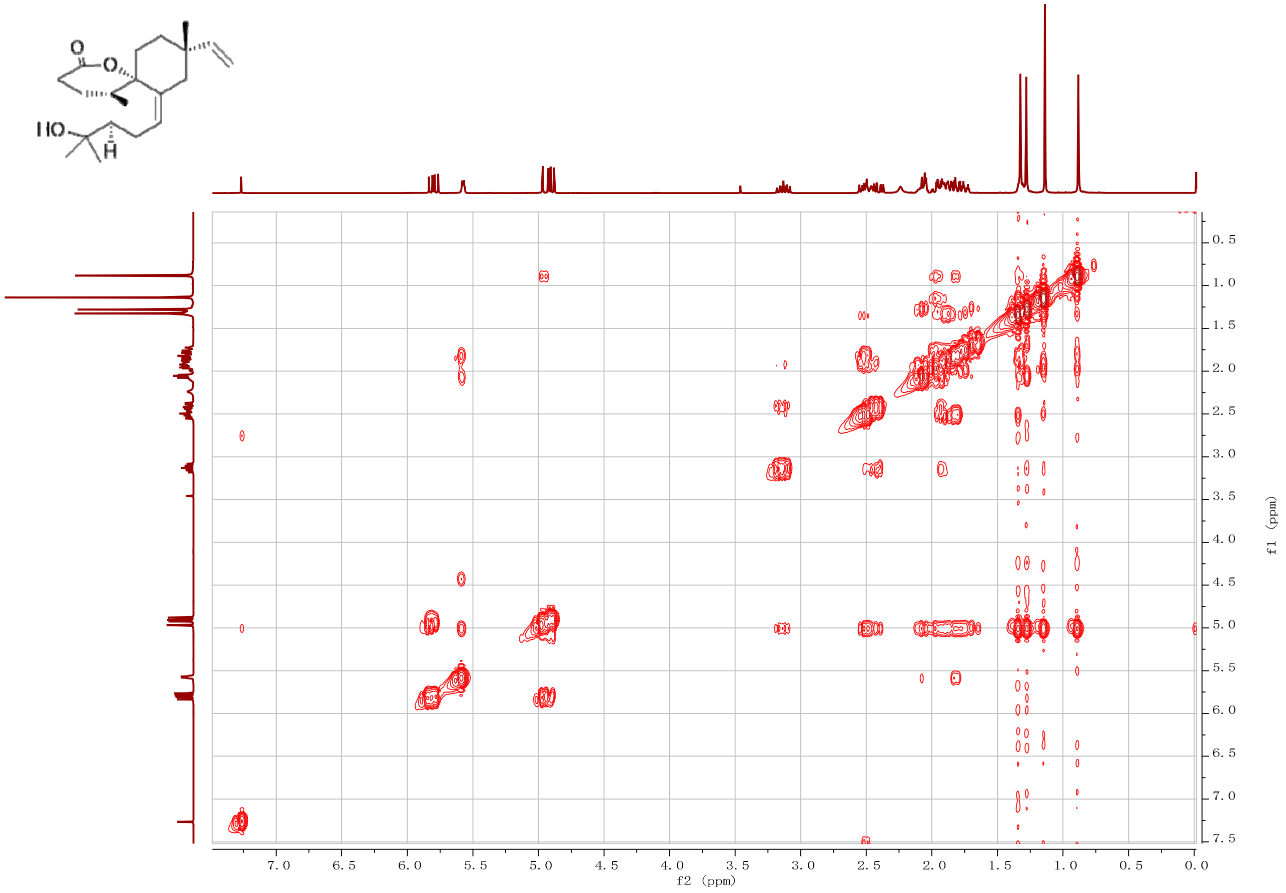

Figure S10. NOESY spectrum of fladin B (**1**) in $CDCl_3$.

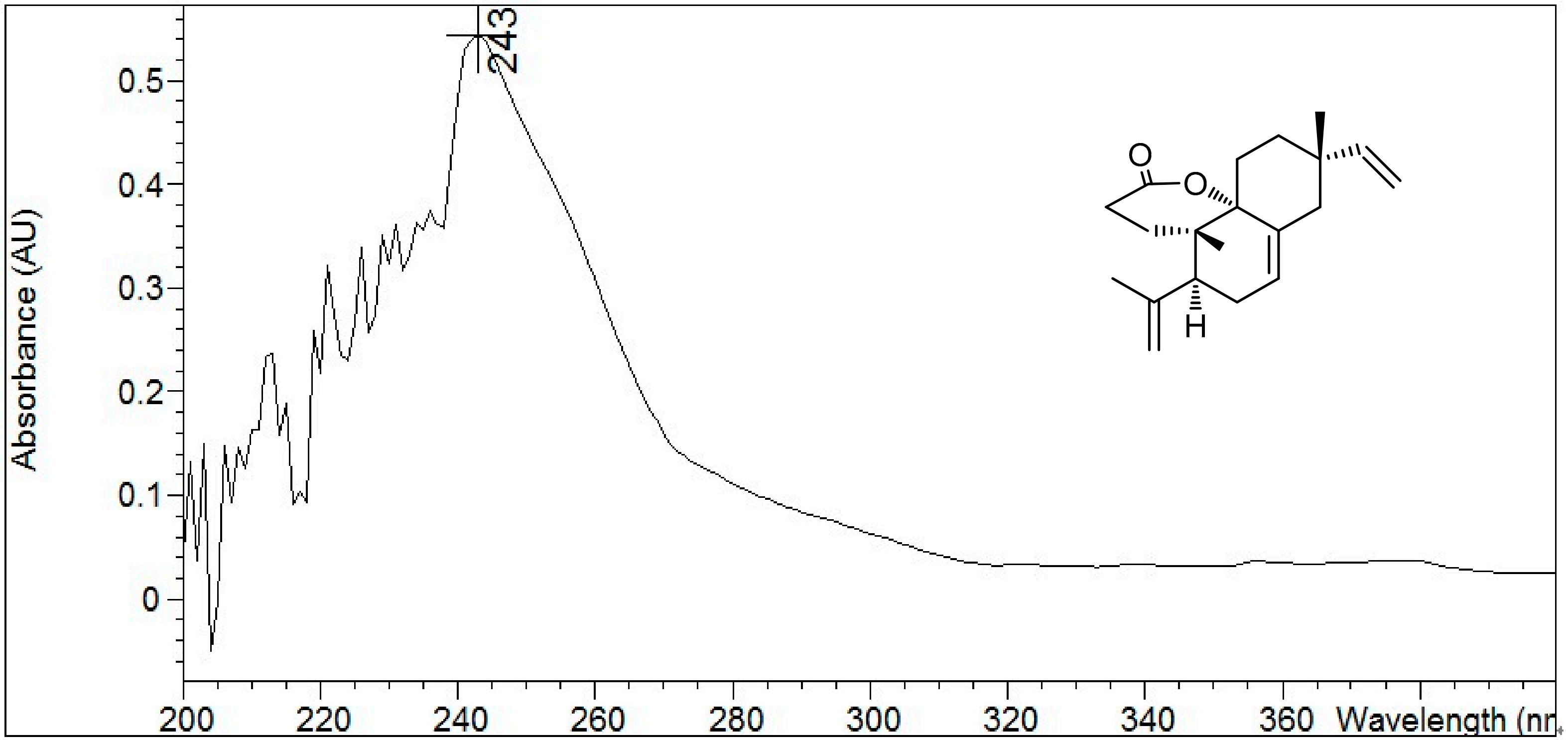

#	Name	Peaks(nm)	Abs(AU)
1	IZ-5	243.0	0.54332

Figure S11. The UV spectrum of fladin C (**2**) in $CHCl_3$.

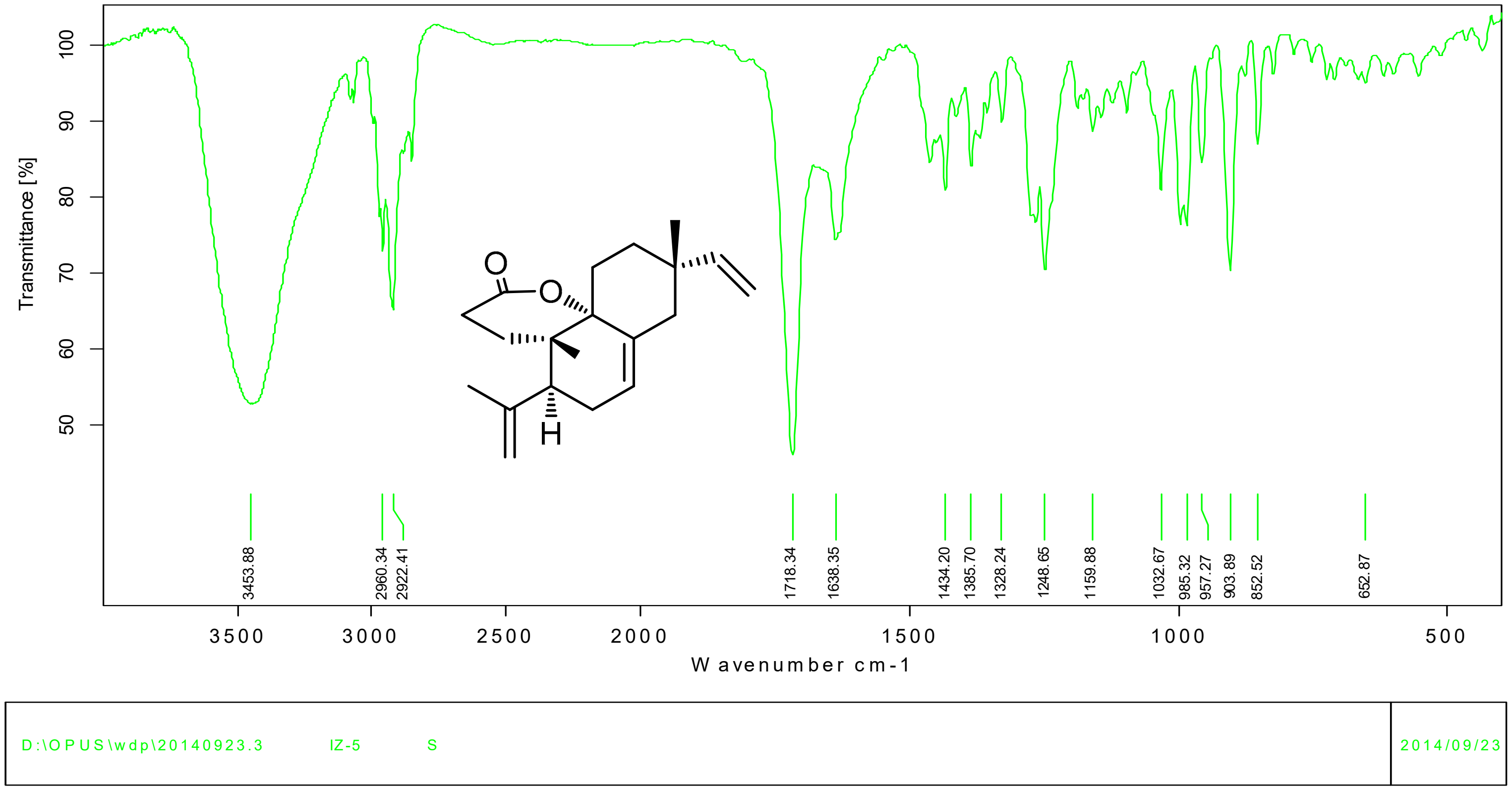

Figure S12. The IR (KBr disc) spectrum of fladin C (**2**).

Mass Spectrum SmartFormula Report

Analysis Info

Analysis Name	D:\Data\Zhang HJ\LKL\IZ-5_1-D,2_01_12814.d	Acquisition Date	10/17/2014 3:45:20 PM
Method	wide_pos (13-05-20).m	Operator	CMED
Sample Name	IZ-5	Instrument / Ser#	micrOTOF-Q 19
Comment			

Acquisition Parameter

Source Type	ESI	Ion Polarity	Positive	Set Nebulizer	2.5 Bar
Focus	Not active			Set Dry Heater	180 °C
Scan Begin	50 m/z	Set Capillary	4500 V	Set Dry Gas	8.0 l/min
Scan End	1600 m/z	Set End Plate Offset	-500 V	Set Divert Valve	Source

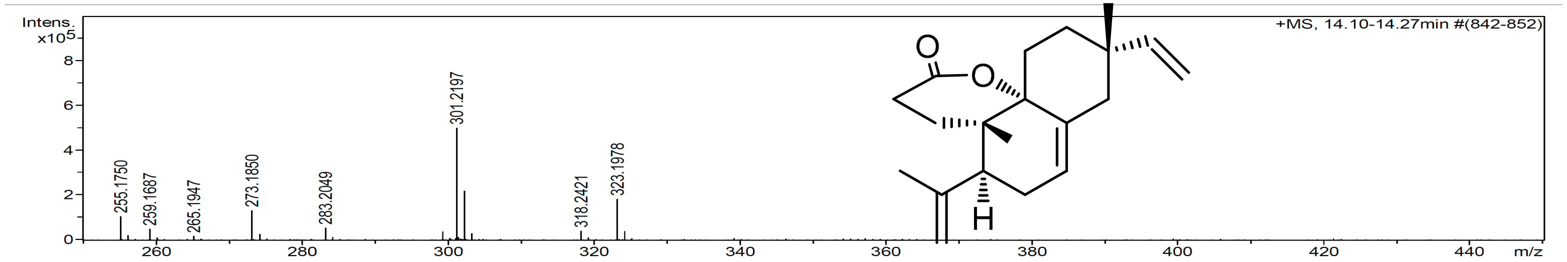

Meas. m/z	#	Formula	m/z	err [ppm]	Mean err [ppm]	rdb	N-Rule	e¯ Conf	mSigma	Std I	Std Mean m/z	Std I VarNorm	Std m/z Diff	Std Comb Dev
301.2197														
	1	C 20 H 29 O 2	301.2162	-11.7	-8.4	6.5	ok	even	128.11	0.1797	0.0029	0.0731	0.0028	0.8183
	2	C 14 H 29 N 4 O 3	301.2234	12.3	15.5	2.5	ok	even	157.35	0.2316	0.0049	0.0925	0.0027	0.8954
	3	C 10 H 25 N 10 O	301.2207	3.4	6.5	3.5	ok	even	170.22	0.2589	0.0024	0.1047	0.0026	0.8366
323.1978														
	1	C 20 H 28 Na O 2	323.1982	1.2	1.2	6.5	ok	even	5.41	0.0087	0.0004	0.0036	0.0001	0.5620
	2	C 18 H 23 N 6	323.1979	0.3	0.3	10.5	ok	even	6.23	0.0089	0.0001	0.0046	0.0000	0.5099
	3	C 16 H 24 N 6 Na	323.1955	-7.1	-7.2	7.5	ok	even	8.42	0.0167	0.0023	0.0077	0.0000	0.8427
	4	C 17 H 27 N 2 O 4	323.1965	-3.8	-3.8	5.5	ok	even	9.08	0.0154	0.0012	0.0063	0.0001	0.7525
	5	C 22 H 27 O 2	323.2006	8.6	8.6	9.5	ok	even	18.09	0.0302	0.0028	0.0125	0.0001	0.9066
	6	C 15 H 28 N 2 Na O 4	323.1941	-11.3	-11.2	2.5	ok	even	21.73	0.0385	0.0036	0.0155	0.0001	0.9411
	7	C 13 H 23 N 8 O 2	323.1938	-12.2	-12.2	6.5	ok	even	21.89	0.0404	0.0039	0.0163	0.0000	0.9486

Bruker Compass DataAnalysis 4.0 printed: 10/21/2014 1:21:54 PM Page 1 of 1

Figure S13. The HR-ESI-MS spectrum of fladin C (**2**).

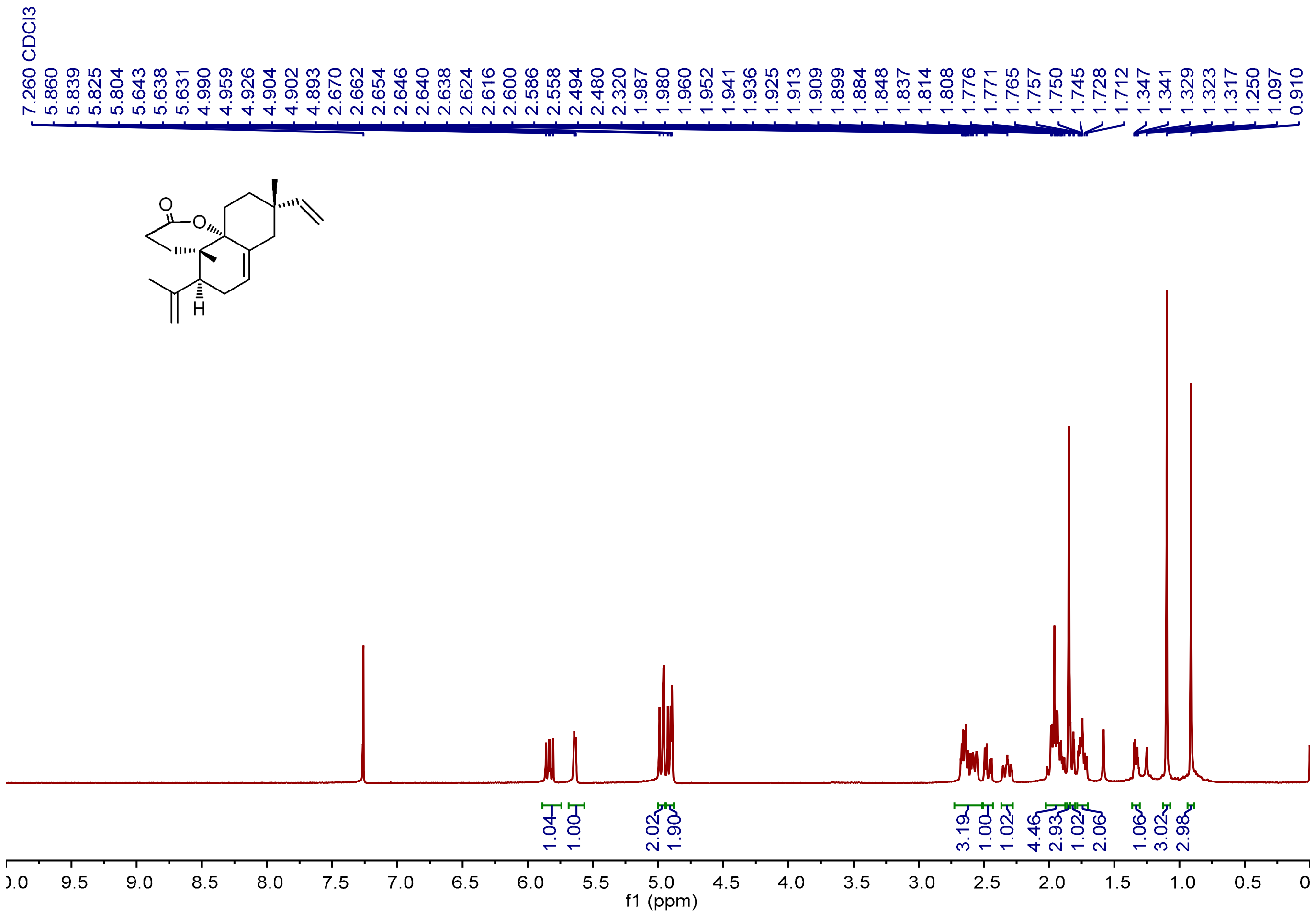

Figure S14. ^{1}H NMR spectrum of fladin C (**2**) in $CDCl_3$ (500 MHz).

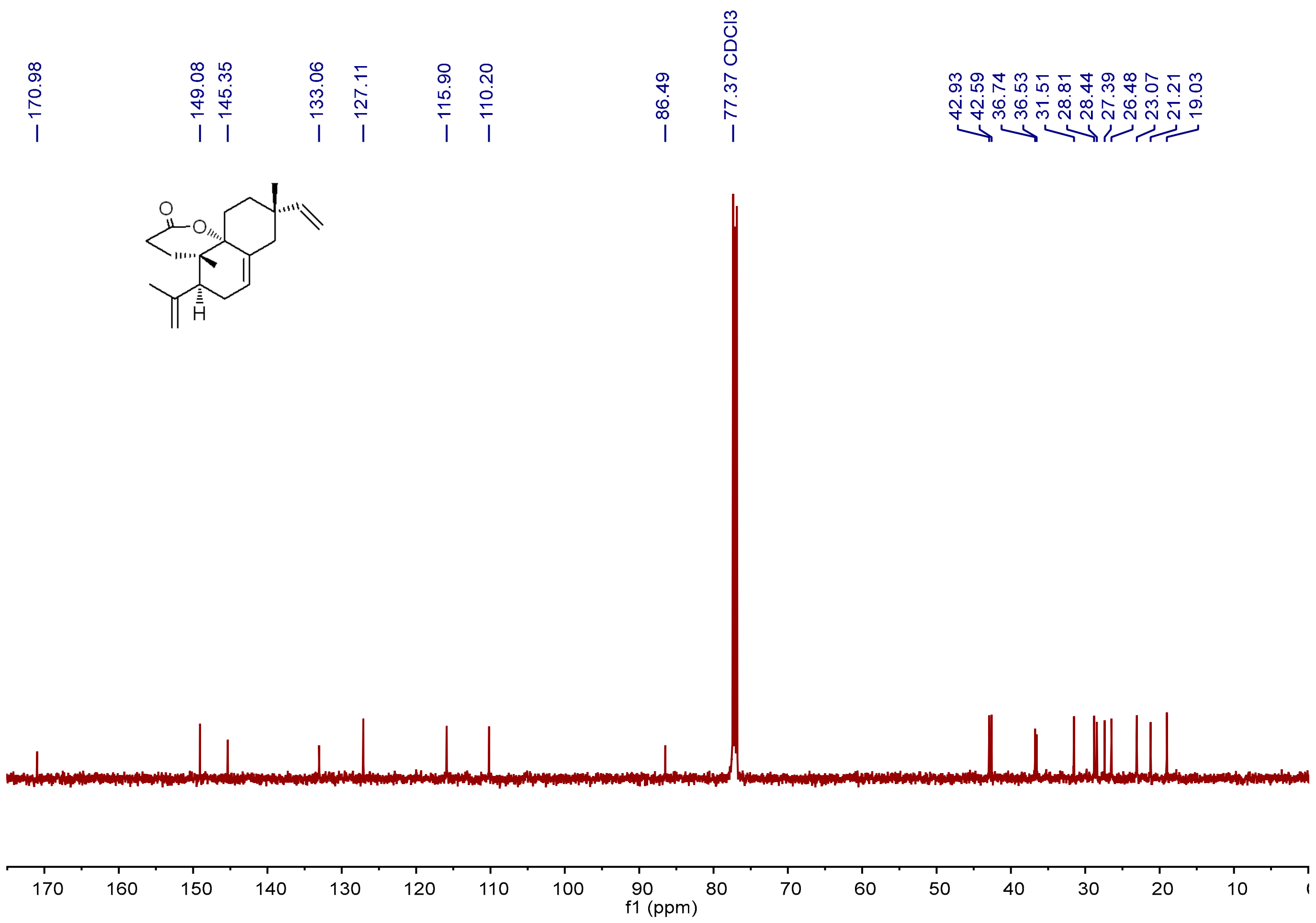

Figure S15. ^{13}C NMR spectrum of fladin C (**2**) in $CDCl_3$ (125 MHz).

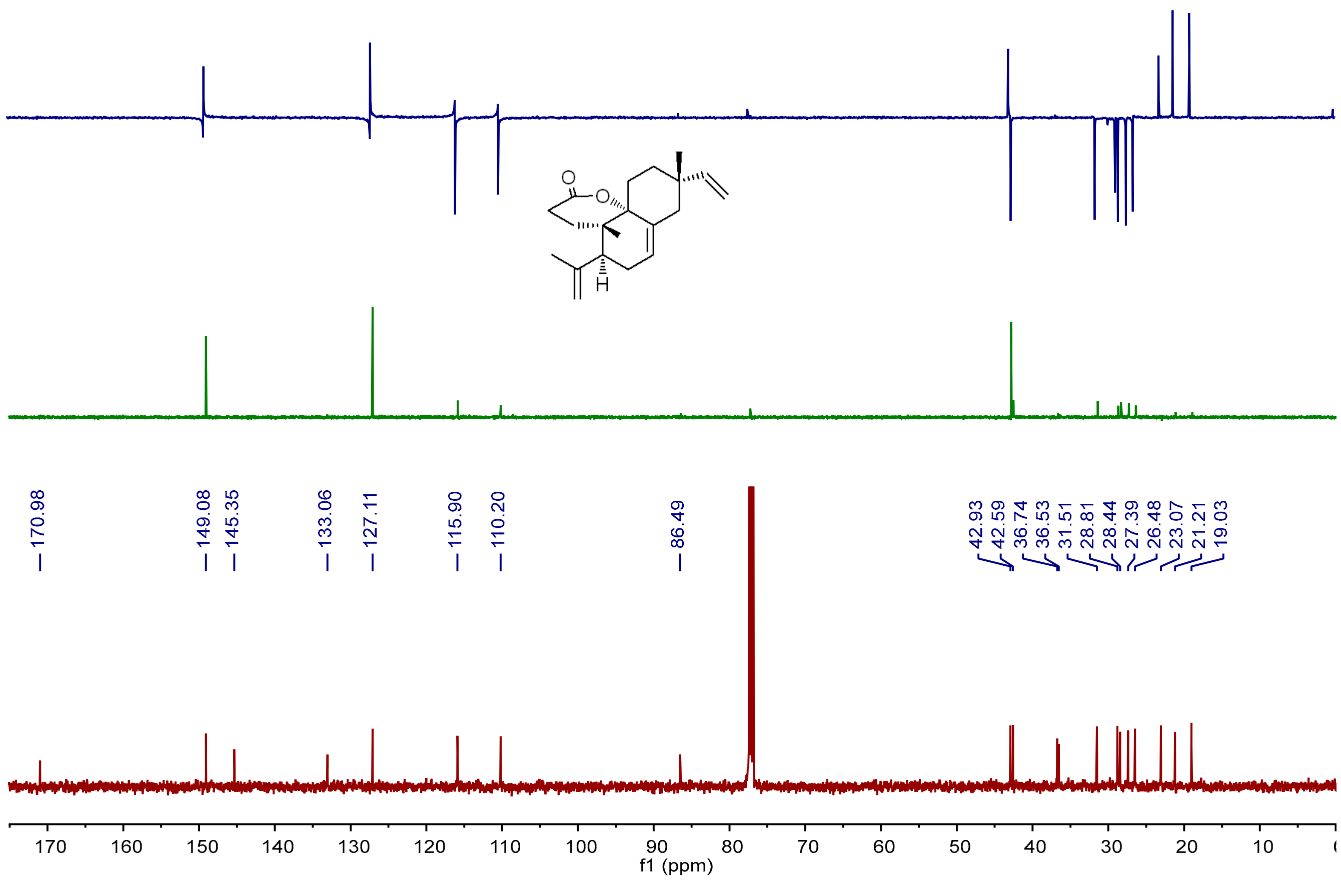

Figure S16. DEPT spectra of fladin C (**2**) in $CDCl_3$.

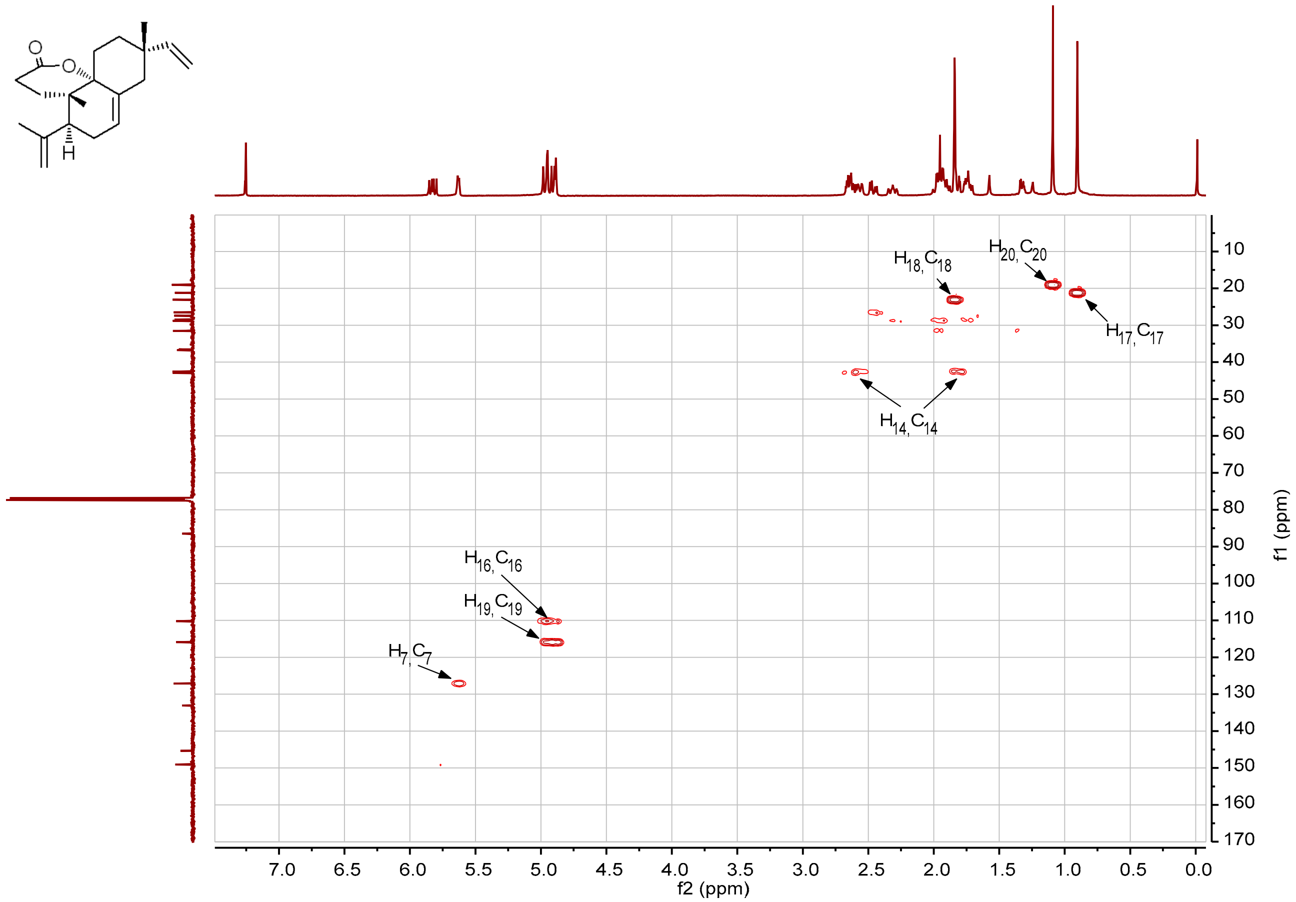

Figure S17. HSQC spectrum of fladin C (**2**) in $CDCl_3$.

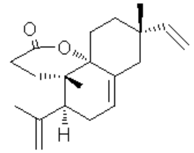

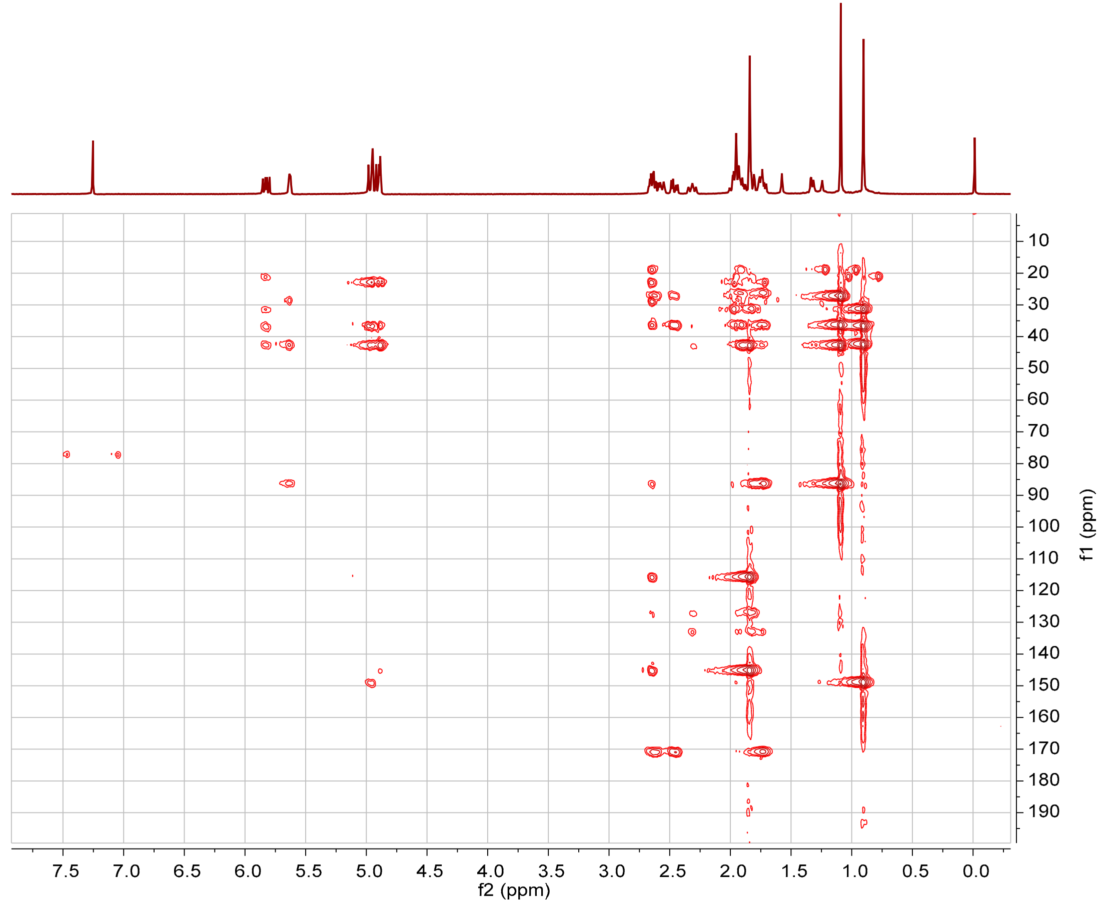

Figure S18. HMBC spectrum of fladin C (**2**) in $CDCl_3$.

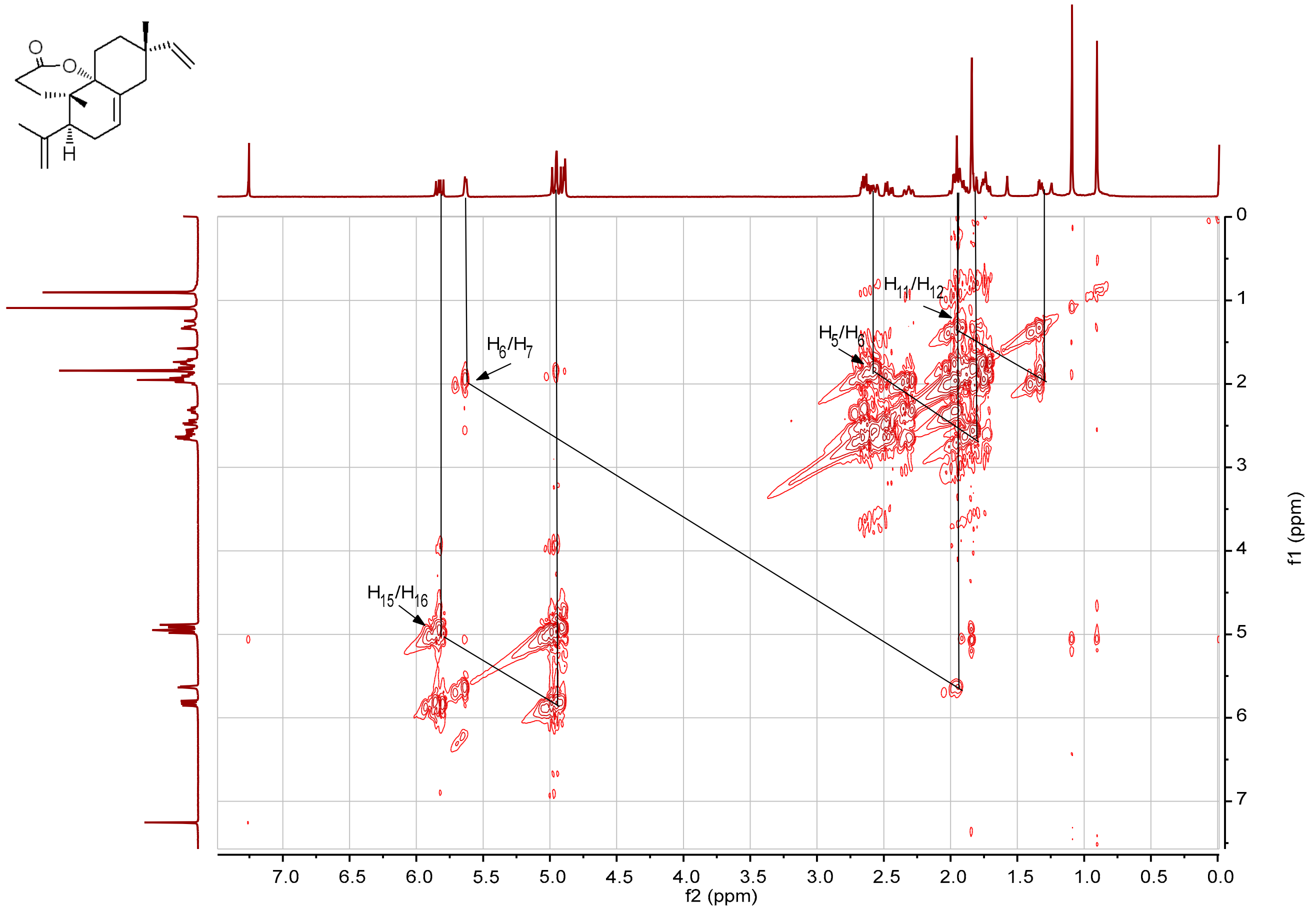

Figure S19. ^{1}H-^{1}H COSY spectrum of fladin C (**2**) in $CDCl_3$.

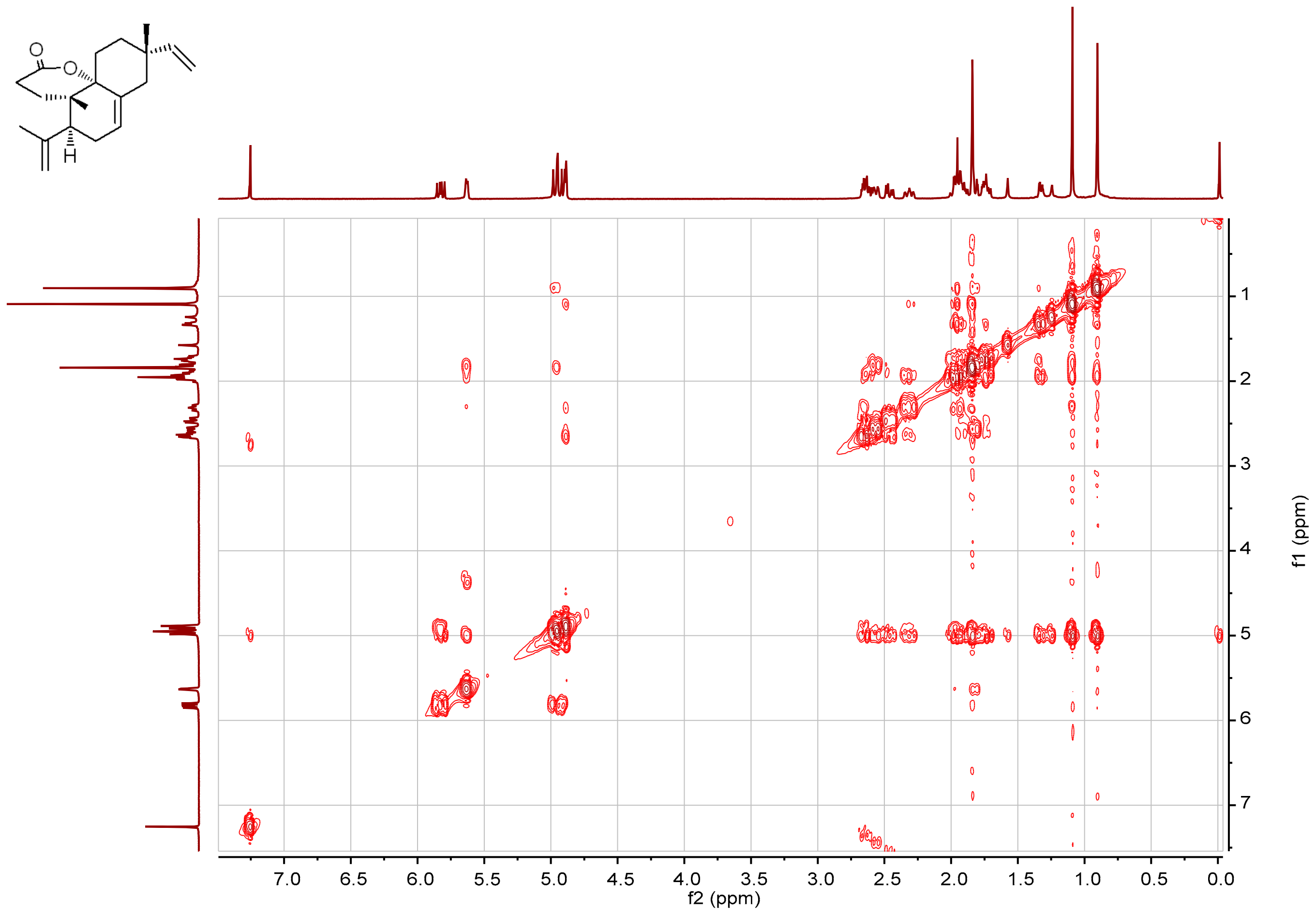

Figure S20. NOESY spectrum of fladin C (**2**) in $CDCl_3$.

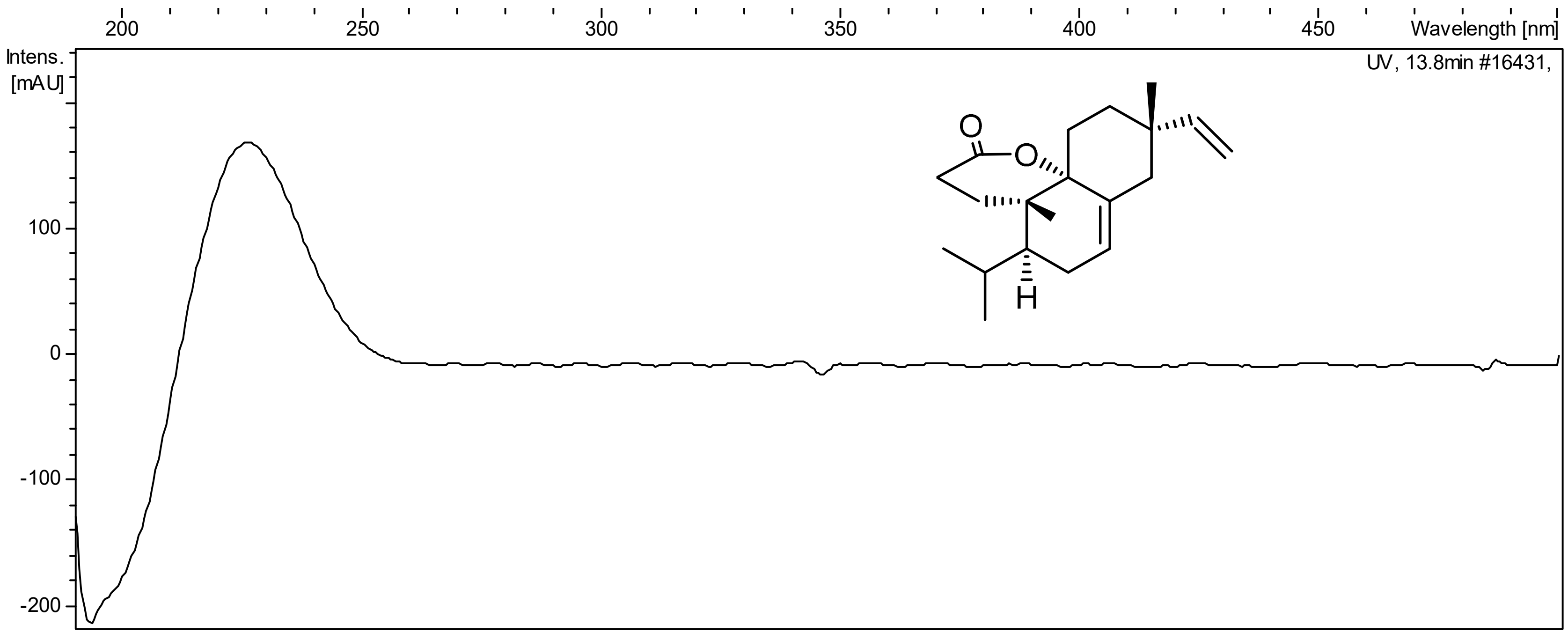

Figure S21. The UV spectrum of fladin D (**3**) in $CHCl_3$.

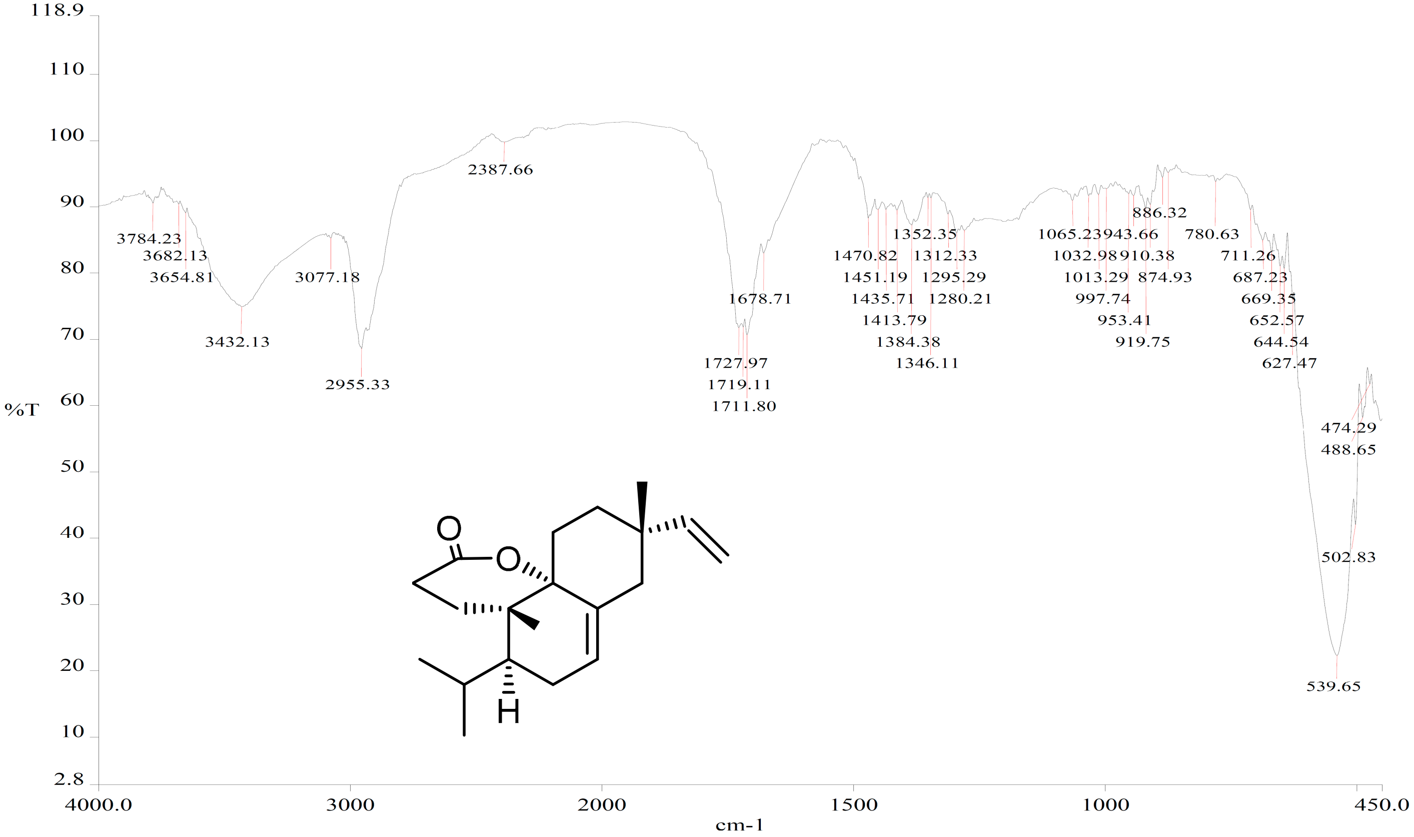

Figure S22. The IR (KBr disc) spectrum of fladin D (**3**).

Compound Mass Spectrum Deconvolution Report

Analysis Info

Analysis Name	D:\Data\alan\Zhang HJ\17-09-06\IZ-X-2_2-D,5_01_17777.d	Acquisition Date	9/6/2017 11:05:46 AM
Method	wide_pos (16-06-06).m	Operator	CMED
Sample Name	IZ-X-2	Instrument / Ser#	micrOTOF-Q 19
Comment			

Acquisition Parameter

Source Type	ESI	Ion Polarity	Positive	Set Nebulizer	2.5 Bar
Focus	Not active	Set Capillary	3500 V	Set Dry Heater	180 °C
Scan Begin	50 m/z	Set End Plate Offset	-500 V	Set Dry Gas	8.0 l/min
Scan End	2200 m/z	Set Collision Cell RF	180.0 Vpp	Set Divert Valve	Source

+MS, 15.1min #899

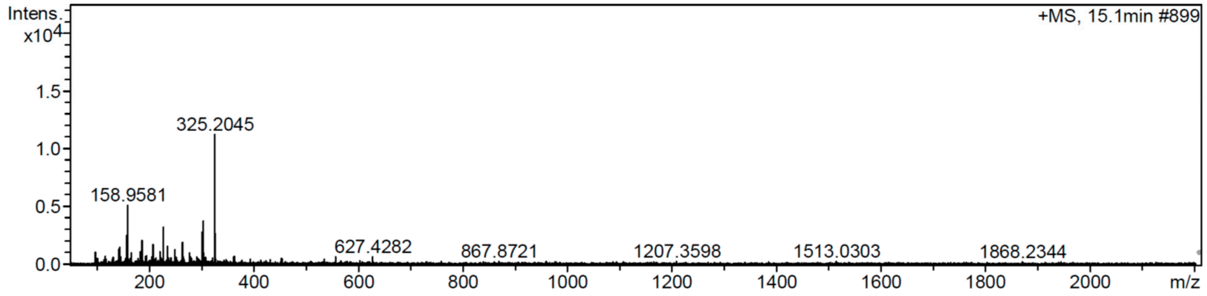

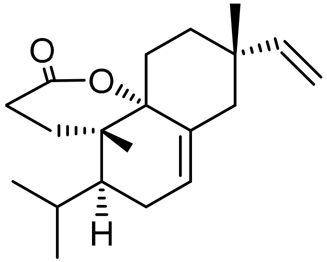

Component	Molecular Mass	Molecule	Absolute Abundance	Relative Abundance

Figure S23. The HR-ESI-MS spectrum of fladin D (**3**).

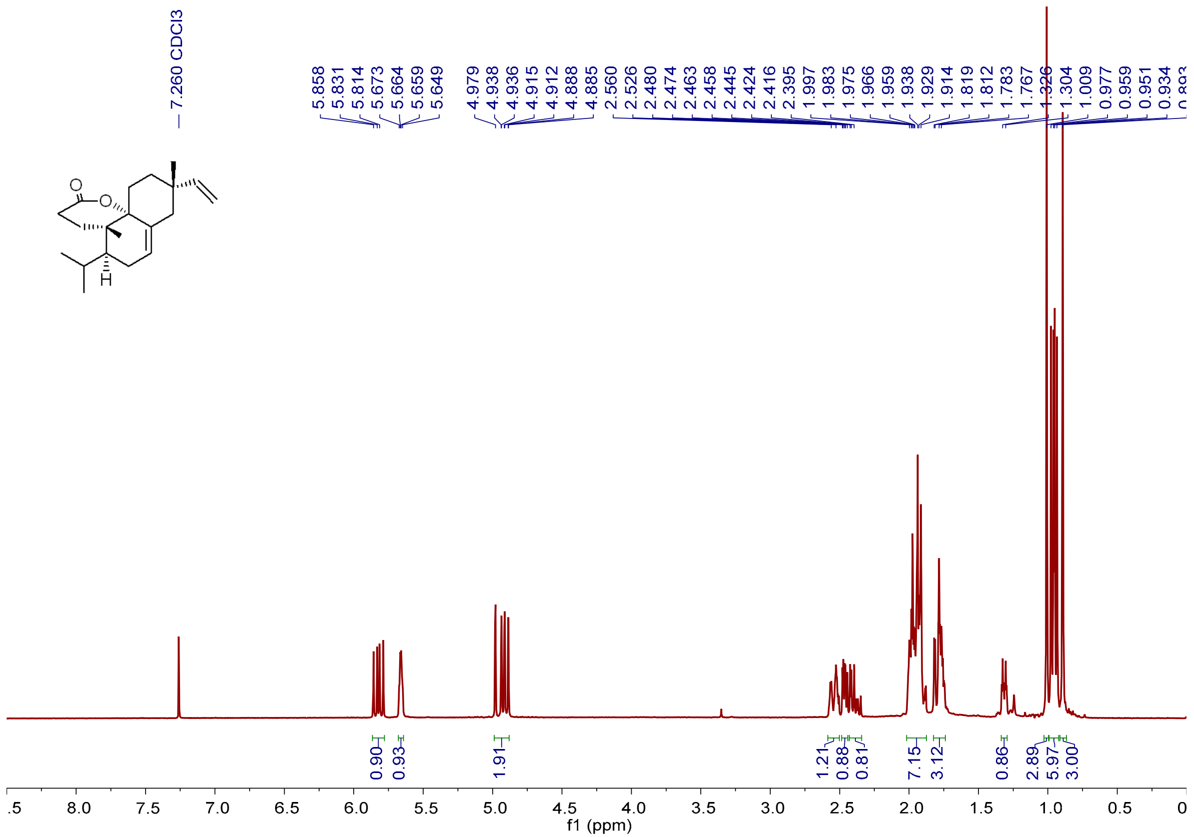

Figure S24. ^{1}H NMR spectrum of fladin D (**3**) in $CDCl_3$ (400 MHz).

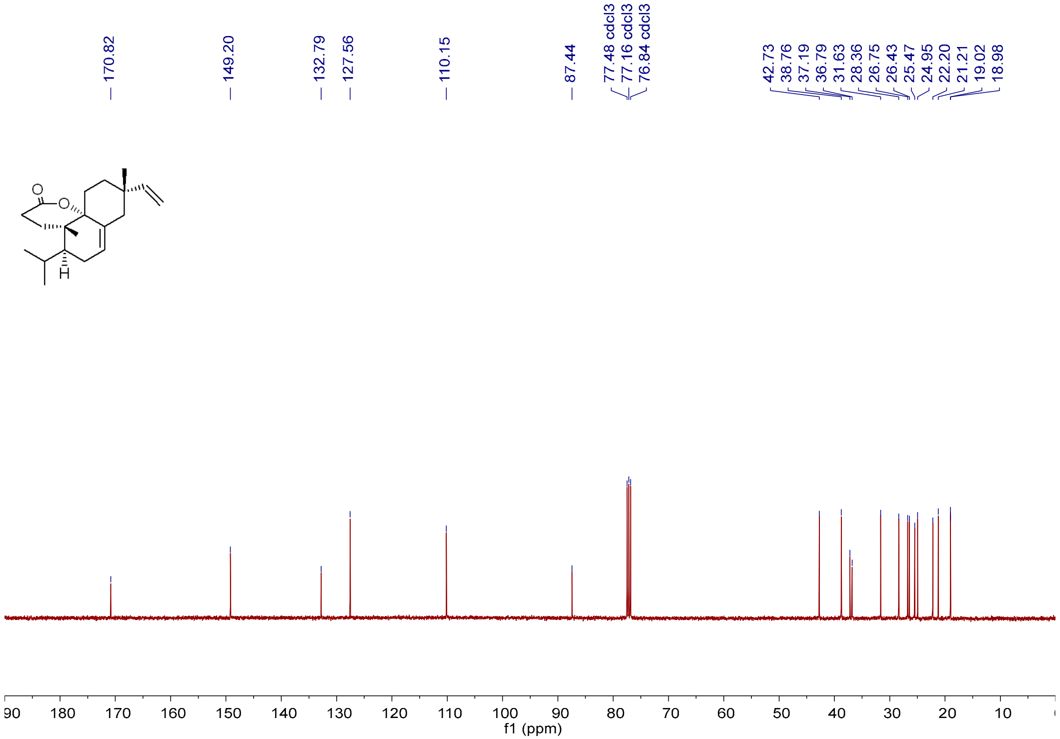

Figure S25. ^{13}C NMR spectrum of fladin D (**3**) in $CDCl_3$ (100 MHz).

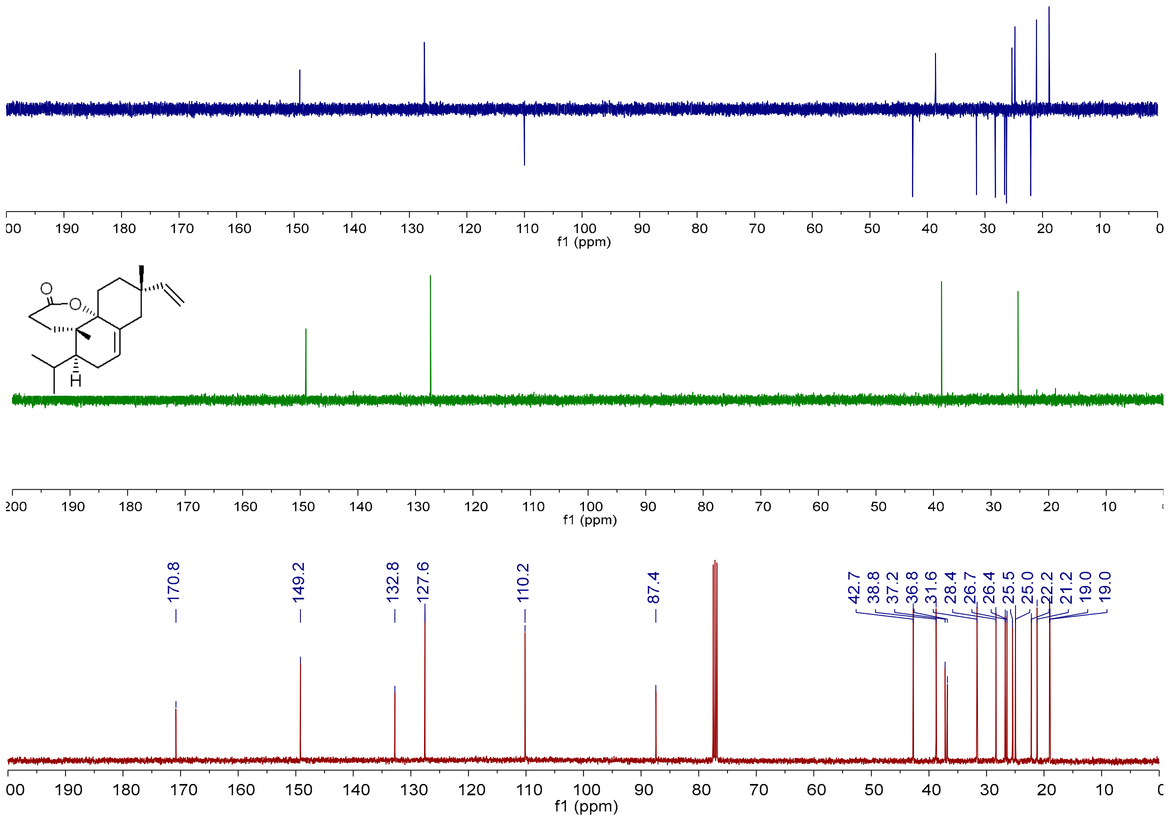

Figure S26. DEPT spectra of fladin D (**3**) in $CDCl_3$.

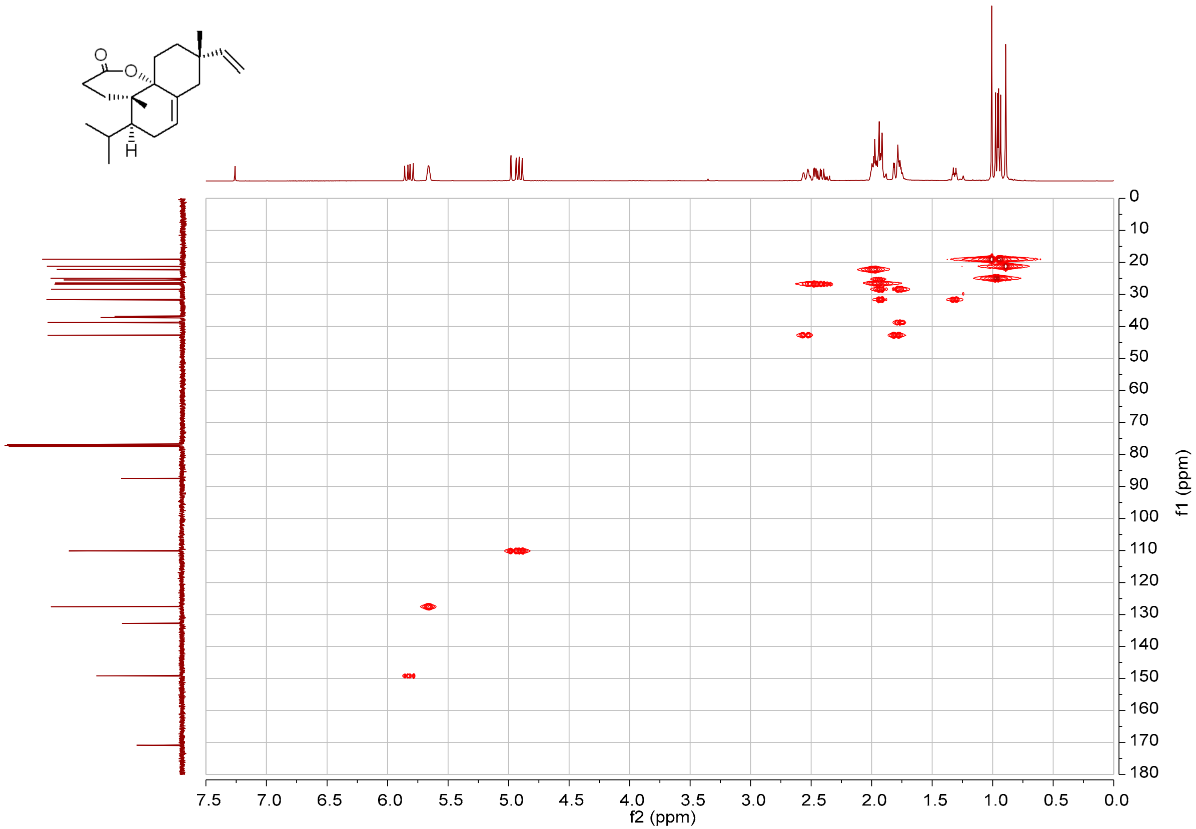

Figure S27. HSQC spectrum of fladin D (**3**) in $CDCl_3$.

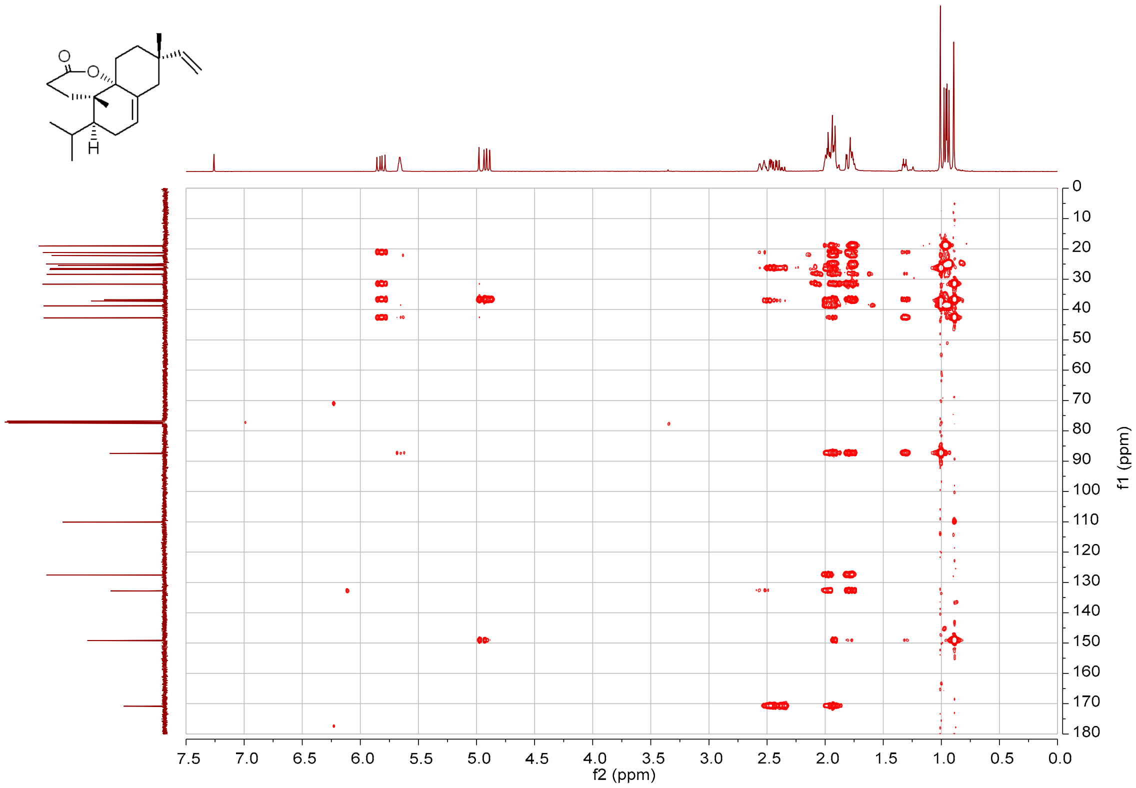

Figure S28. HMBC spectrum of fladin D (**3**) in $CDCl_3$.

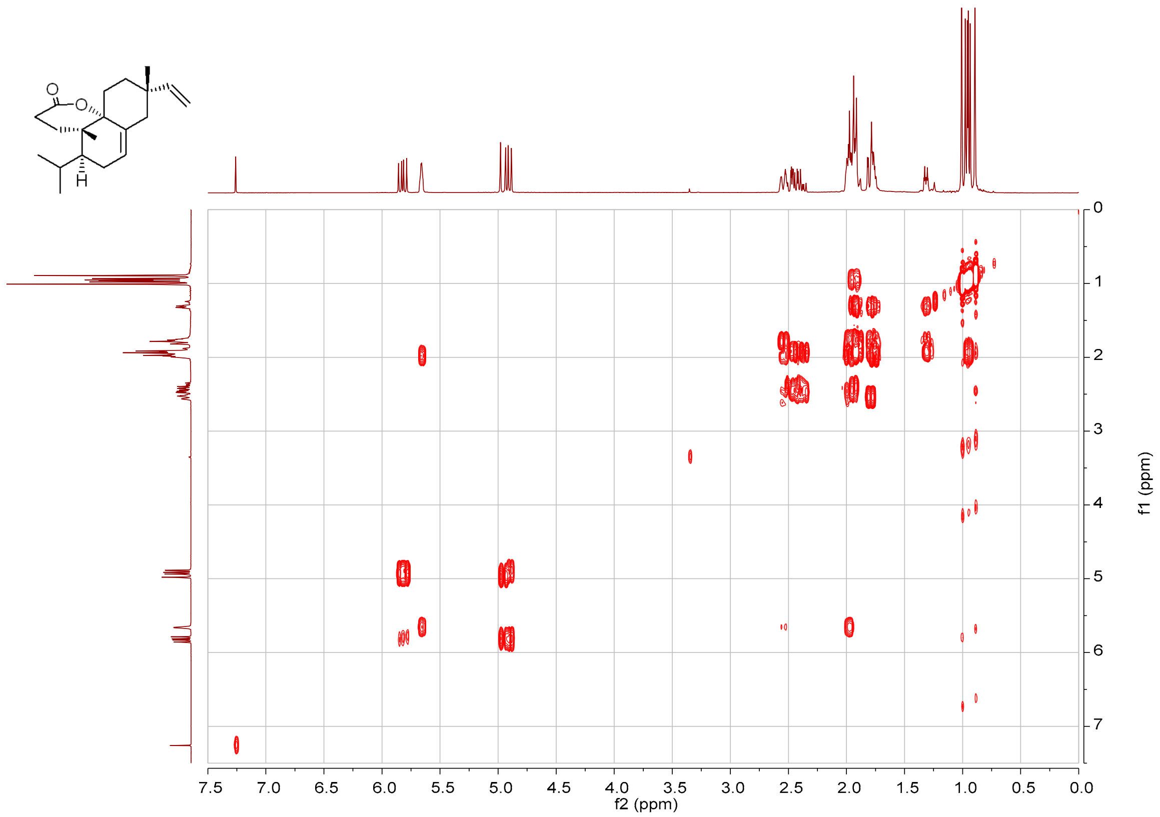

Figure S29. ^{1}H-^{1}H COSY spectrum of fladin D (**3**) in $CDCl_3$.

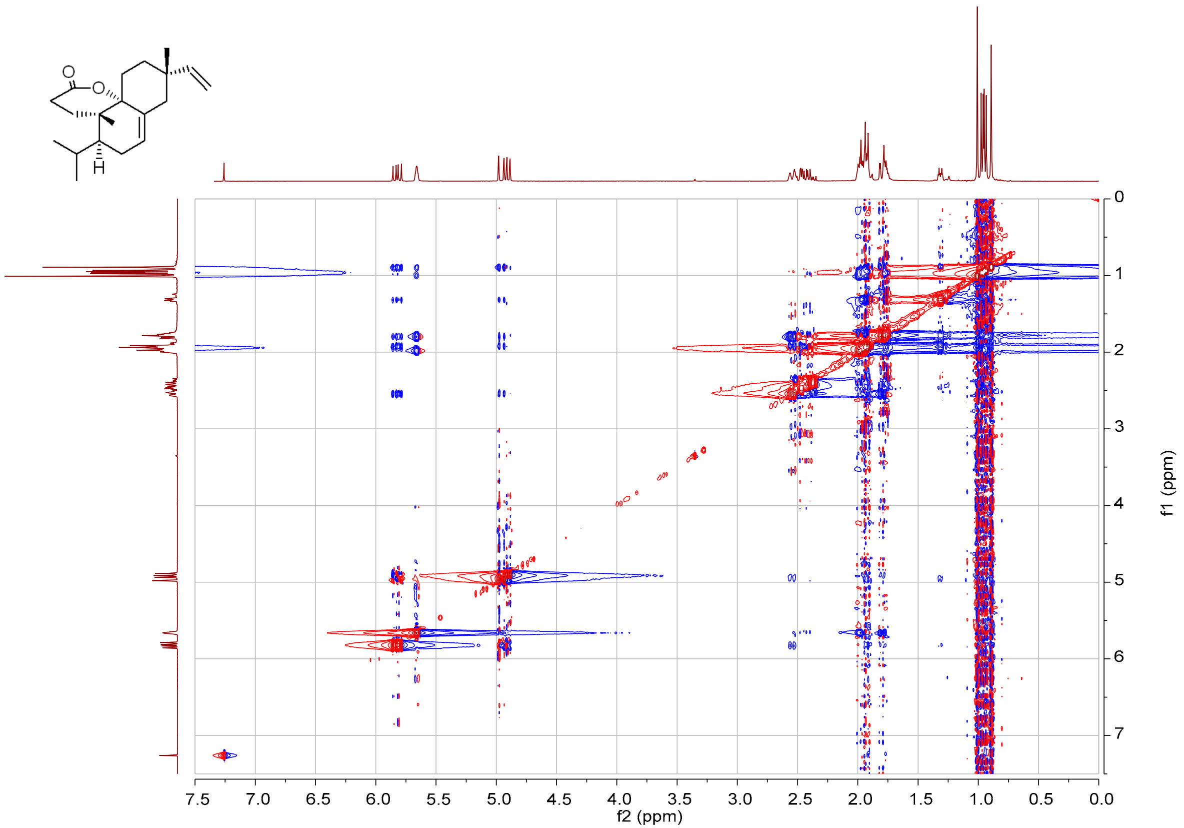

Figure S30. NOESY spectrum of fladin D (**3**) in $CDCl_3$.
